# Identification of Key Biomarkers in Bladder Cancer: Evidence from a Bioinformatics Analysis

**DOI:** 10.3390/diagnostics10020066

**Published:** 2020-01-24

**Authors:** Chuan Zhang, Mandy Berndt-Paetz, Jochen Neuhaus

**Affiliations:** Department of Urology, University of Leipzig, 04103 Leipzig, Germany; Chuan.Zhang@medizin.uni-leipzig.de (C.Z.); Mandy.Berndt@medizin.uni-leipzig.de (M.B.-P.)

**Keywords:** bladder cancer, bioinformatics analysis, differentially expressed genes, TCGA-BLCA database, GEO databases

## Abstract

Bladder cancer (BCa) is one of the most common malignancies and has a relatively poor outcome worldwide. However, the molecular mechanisms and processes of BCa development and progression remain poorly understood. Therefore, the present study aimed to identify candidate genes in the carcinogenesis and progression of BCa. Five GEO datasets and TCGA-BLCA datasets were analyzed by statistical software R, FUNRICH, Cytoscape, and online instruments to identify differentially expressed genes (DEGs), to construct protein‒protein interaction networks (PPIs) and perform functional enrichment analysis and survival analyses. In total, we found 418 DEGs. We found 14 hub genes, and gene ontology (GO) analysis revealed DEG enrichment in networks and pathways related to cell cycle and proliferation, but also in cell movement, receptor signaling, and viral carcinogenesis. Compared with noncancerous tissues, TPM1, CRYAB, and CASQ2 were significantly downregulated in BCa, and the other hub genes were significant upregulated. Furthermore, MAD2L1 and CASQ2 potentially play a pivotal role in lymph nodal metastasis. CRYAB and CASQ2 were both significantly correlated with overall survival (OS) and disease-free survival (DFS). The present study highlights an up to now unrecognized possible role of CASQ2 in cancer (BCa). Furthermore, CRYAB has never been described in BCa, but our study suggests that it may also be a candidate biomarker in BCa.

## 1. Introduction

Bladder cancer (BCa) is one of the most common malignancies, with a high rate of recurrence, and involves associated high morbidity and mortality, especially in advanced BCa [1]. Surgical resection, neoadjuvant chemotherapy, intravesical treatment, radiotherapy, and photodynamic therapy (PDT) are conventional therapeutic approaches to BCa [2,3,4]. According to the clinical spectrum of BCa, it is significant to explore the disease mechanisms, and to identify precise and effective biomarkers for early diagnosis of BCa with no significant clinical symptoms, for evaluating prognosis, and for developing effective strategies of BCa treatment.

Bladder cancer is mostly induced by exposure to toxic substances, and smoking is the leading risk factor. A papillary and a nonpapillary form of BCa are distinguished. Based on the heritage from different progenitor cells, those two forms lead to various molecular subtypes, with different clinical behavior. The superficial, luminal papillary tumors are genetically stable, remain noninvasive and nonmetastatic, and can be treated curatively by repeated transurethral resection. The invasive form derives from a different urothelial precursor, progresses to invasion of the bladder wall and metastasis [5]. Evidence of different basic molecular mechanisms comes from mouse models. Mutant ras genes induced urothelial hyperplasia at low copy numbers and papillary tumors at high copy numbers, while inactivation of the tumor protein p53 and retinoblastoma (RB1) pathways seem to induce carcinoma in situ (CIS) tumors, able to progress into invasive BCa [6,7]. Ras activation is coupled to the Wnt signaling β-catenin pathway, driving bladder tumorigenesis [8]. P53 alterations are involved in tumor progression to more aggressive forms [9] and the RB1 pathway plays a critical role in the regulation of the cell cycle and cell death [10,11]. Until now, there has been no consensus about the use of urinary markers or tests for non-muscle-invasive bladder cancer (NMIBC) from the international panels on bladder cancer [12,13,14]. While the European Association of Urology (EAU) and the Canadian Urological Association (CUA) do not recommend any urinary test in their guidelines, the American Urological Association (AUA), the National Comprehensive Cancer Network (NCCN), and the National Institute for Health and Care Excellence (NICE) recommend the use of certain available tests (UroVysion, ImmunoCyt NMP22) under certain patient conditions [12].

In recent decades, microarray technology and bioinformatics have been widely used to identify the differentially expressed genes (DEGs) and functional pathways involved in cancers [15]. However, in independent microarray analysis, the false positive rates and the small number of samples were predominant factors that limited the ability to obtain reliable results. In the present in silico analysis, we used six public access databases, the Cancer Genome Atlas (TCGA) and five mRNA microarray datasets from the Gene Expression Omnibus (GEO), to detect DEGs in bladder cancer tissues and noncancerous bladder tissues (further nominated controls). Subsequently, we performed gene ontology (GO) enrichment analysis, Kyoto Encyclopedia of Genes and Genomes (KEGG) pathway analysis, protein‒protein interaction (PPI) network analysis, and gene co-expression network analysis to identify protein-coding genes and related pathways potentially playing an essential role in BCa. We did not analyze noncoding RNA in the present study.

**Figure 1:**
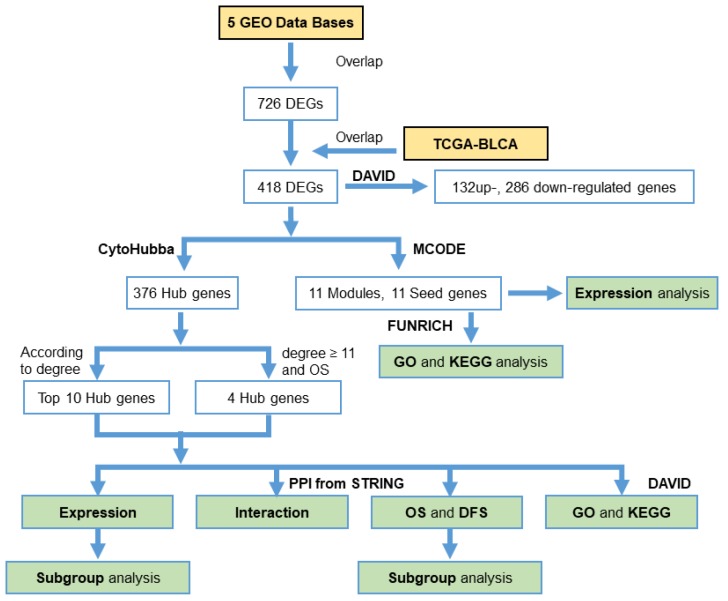
Study design. Workflow of the analysis steps and software used.

For stepwise data reduction, we only used overlapping DEGs from the five GEO databases in the first place and in a second step only pursued those DEGs overlapping with the TCGA-BLCA database. Based on those DEGs, we identified hub genes using CytoHubba software and then used the top 10 hub genes according to the reported degree of connection and another four clearly cancer-related hub genes for detailed analyses regarding their association with overall survival (OS), disease-free survival (DFS), and involvement in regulatory pathways. Figure 1 summarizes the study design and workflow.

## 2. Materials and Methods

### 2.1. Acquisition of Microarray Data

Five gene expression datasets, GSE27448 [16,17], GSE52519 [18], GSE61615 [19], GSE76211 [20,21] and GSE100926 [22], were downloaded from GEO (http://www.ncbi.nlm.nih.gov/geo) [23]. The annotation information of the datasets and the platforms is shown in Table 1. The probe IDs were converted into corresponding gene symbols. The TCGA-BLCA dataset was downloaded from TCGA (https://portal.gdc.cancer.gov/) [24]. Statistical software R (Version 3.3.2. https://www.r-project.org/) with R studio (Version 1-1-463), and Bioconductor (http://www.bioconductor.org/) packages “pd.hta.2.0”, ”org.Hs.eg.db”, and “tidyr” were utilized to convert the IDs.

### 2.2. Identification of DEGs

First, we validated the quality of the microarray data by using the statistical software R, including the packages “lima”, “affyPLM”, “RColorBrewer”, “affy”, “pd.hta.2.0”, “ggplot2”, and “impute”. We conducted relative log expression (RLE) box plot for quality control. GEO2R (http://www.ncbi.nlm.nih.gov/geo/geo2r) is an interactive web tool used to compare the multiple groups in a GEO series. A |Log FC (Fold Change)| >1 and a *p*-value < 0.05 were considered statistically significant. We used GEO2R to detect DEGs between BCa and noncancerous samples.

### 2.3. GO and KEGG Analyses of DEGs

FUNRICH software (Version 3.1.3) and DAVID (http://david.ncifcrf.gov) (Version 6.7) [25] were used to annotate, visualize, and integrate the data, and to extract the important biological information. KEGG is a database resource for understanding gene functions, linking genomic information with high-level function information [26]. Gene ontology (GO) enrichment analysis is the dominant tool used in bioinformatics to perform enrichment analyses on gene sets, finding up-or downregulation under pathological conditions and identifying the underlying biological process of target genes [27]. We here explored DEGs by GO and KEGG analysis to find the critical biological process, molecular function, and crucial pathways closely related to the initiation and development of BCa. *p* < 0.05 was considered statistically significant.

### 2.4. Protein‒Protein Interaction (PPI) Network Analysis

The PPI network analysis is a crucial way to identify the key genes and relevant gene modules involved in interactions. The search tool for the retrieval of interacting genes (STRING; http://string-db.org) (Version 11.0) [28] was used to acquire the PPI information of the DEGs. Subsequently, Cytoscape software (Version 3.7.2) [29] was performed to construct a PPI network, and to analyze the functional interactions between proteins. Then, we used the plugin tool cytoHubba and molecular complex detection (MCODE) in Cytoscape to elucidate the biological significance of gene modules (subnetworks) in BCa. *p* < 0.05 was considered to have statistical significance.

### 2.5. Clinical Data Analysis and Oncomine Analysis

According to the clinical information from TCGA-BLCA, statistical software R and the packages “ComplexHeatmap”, “clusterProfiler”, ”survival”, ”survMisc”, “survminer”, and “RColorBrewer” were used to analyze the expression levels in different groups, and for survival analysis across different experimental conditions. Moreover, the webservers and analysis tools of gene expression profiling interactive analysis (GEPIA) [30] (http://gepia.cancer-pku.cn) and UALCAN [31] (http://ualcan.path.uab.edu) were used for subgroup analysis. If the sample size was below seven, we excluded the subgroup from the analysis. In addition, Oncomine analysis was performed to analyze the expression of the representative hub genes in different datasets, and to verify the expression level between noncancerous tissues and BCa samples [32]. *p* < 0.05 was considered to have statistical significance. 

## 3. Results

The study design and workflow of the data mining are depicted in Figure 1.

### 3.1. Identification of DEGs in BCa

All datasets (GSE27448, GSE525519, GSE61615, GSE76217, and GSE100926) passed the quality control tests (Figure S1). Subsequently, we identified DEGs through the analysis of microarray results. 

We found 4701 DEGs in GSE27488, 742 in GSE525519, 736 in GSE61615, 658 in GSE76217, 194 in GSE100926, and 2873 in TCGA-BLCA. For data reduction, we compared the database results and focused on the overlapping DEGs indicated in Figure 1.

**Figure 2:**
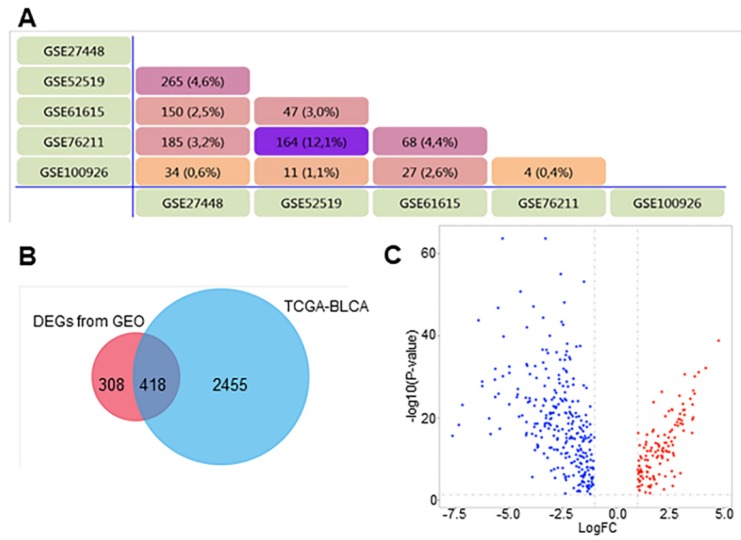
Venn diagram and Volcano plot of DEGs. (**A**) DEGs were selected with |log Fold Change (FC)| > 1 and *p*-value < 0.05 among the mRNA expression profiling sets GSE27448, GSE525519, GSE61615, GSE76217 and GSE100926. (**B**) Overlapped the 726 DEGs with TCGA-BLCA database. (**C**) 132 upregulated genes and 286 significantly downregulated genes in BCa samples. Upregulated genes are marked in red; downregulated genes are marked in blue.

The five GEO datasets showed considerable overlap: 726 genes were found in at least two GEO datasets (Figure 2A). Comparison with the TCGA-BLCA data revealed an overlap of 418 DEGs of the 726 DEGs identified in the GEO datasets, excluding the genes overlapping with the 2873 DEGs found in the TCGA-BLCA database (Figure 2B). Those 418 DEGs were selected for further analyses, consisting of 132 significantly upregulated genes and 286 significantly downregulated genes (Figure 2C).

### 3.2. GO and KEGG Enrichment Analyses of DEGs

We analyzed the gene set of 418 DEGs (132 upregulated; 286 downregulated) using DAVID 6.8. In the GO analysis we focused on the categories of biological process (BP), cellular component (CC), and molecular function (MF), listing the top 10 hits. In the KEGG analysis, we also classified the pathways upon gene counts and the *p*-value.

#### 3.2.1. GO Biological Process (BP)

Based on the 418 DEGs, the GO analysis reported in total 950 chart records in the category BP. “Cell division” was the most significant term according to the adjusted *p*-value after the Benjamini—Hochberg procedure, followed by “mitotic cell cycle process”, “mitotic cell cycle”, and other cell cycle-associated terms (the top 10 GO terms in Table S1). We further analyzed the up- and downregulated genes in selected terms with relevance to cancer (“cytoskeleton organization”, “nuclear division”, “organelle fission”, “cytokinesis”, and “regulation of actin filament-based process”).

Upregulated gene enrichment analysis revealed significant DEGs enrichment in the cell cycle networks: “cell cycle”, “cell cycle process”, “mitotic cell cycle”, “mitotic cell cycle process”, “regulation of cell cycle.” The downregulated DEGs enriched in cancer-related terms were: “system process”, “regulation of signaling”, “regulation of cell communication”, “regulation of molecular function”, and “cellular response to chemical stimulus” (Table S1).

#### 3.2.2. GO Cellular Component (CC) 

Based on the 418 DEGs, the GO_CC analysis revealed 117 chart records. The most significant terms according to the adjusted *p*-value after the Benjamini‒Hochberg procedure were “Contractile fiber”, “Myofibril”, and other cytoskeleton-associated terms (Table S2). Upregulated DEGs were enriched in “nucleoplasm”, “cytosol”, “microtubule cytoskeleton”, and other terms related to cytoskeletal function including nuclear function (Table S2). Interestingly, the downregulated DEGs dominated in “extracellular region”, “membrane-bounded vesicle”, “extracellular vesicle”, “cell junction”, and other cell communication-associated terms (Table S2).

#### 3.2.3. GO Molecular Function (MF)

Based on the total 418 DEGs, GO_MF analysis revealed 88 chart records. Cytoskeleton-associated terms were the most significant: “cytoskeletal protein binding”, “acting binding”, “microtubule binding”, and “tubulin binding” (Table S3). Upregulated DEGs were enriched in terms related to signaling pathway function: “heterocyclic compound binding”, “organic cyclic compound binding”, “nucleotide binding”, nucleoside phosphate binding, and others, while downregulated DEGs were enriched in “ion binding”, “cation binding”, and other receptor- and second messenger-associated terms (Table S3).

#### 3.2.4. KEGG Pathway Analysis

The most prominent KEGG pathway was “pathways in cancer” with 21 genes, followed by “cell cycle” with 18 counts and “focal adhesion” with 13 counts. Table S4 shows the highest scoring pathways from 10‒21 gene counts. The KEGG pathways were roughly in line with the major GO terms found in the GO analyses confirming the validity of the analytical approach. Most upregulated DEGs related to the cancer relevant “cell cycle” and “p53 signaling pathway”, but the third-highest number of DEGs were enriched in “viral carcinogenesis.” On the other hand, downregulated DEGs were associated with “pathways in cancer”, “cGMP-PKG signaling pathway”, and other signaling pathways, but also “focal adhesion” and “regulation of actin cytoskeleton” (Table S4).

### 3.3. PPI Network Analysis

**Figure 3:**
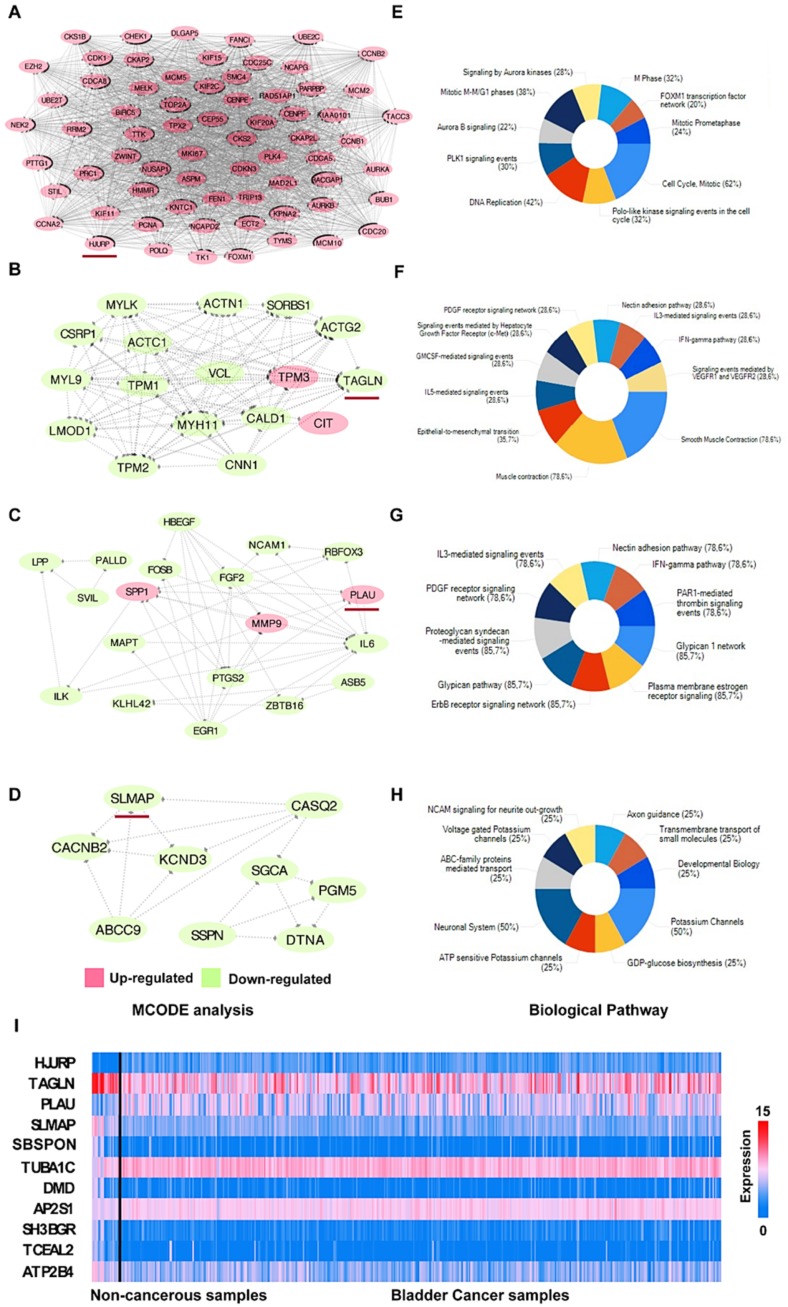
PPI network and biological pathway analysis for modules. (**A**‒**D**) PPI network of the top four significant modules: the seed gene in each module is marked with a red underline; a label in red means the gene is upregulated, while a label in green means it is downregulated. (**E**‒**H**) The top 10 biological pathways of each of the four modules. (**I**) Hierarchical clustering of 11 seed genes. The samples on the left of the black bar are noncancerous bladder tissue (regarded as controls) from the TCGA-BLCA dataset (*n* = 19) and the samples on the right of the black bar are BCa samples from the TCGA-BLCA dataset (*n* = 406). Upregulated genes are marked in red; downregulated genes are marked in blue.

According to the outcomes of the STRING analysis, we constructed the PPI network of DEGs using Cytoscape software. The network consisted of 414 nodes and 3592 edges, the average local clustering coefficient was 0.465 and the PPI enrichment *p*-value < 1.0 × 10^−16^. We used the plugin MCODE in Cytoscape to identify 11 important modules. The seed genes of the top four significant modules, depicted in Figure 3A‒H, were HJURP (Holliday junction recognition protein; Module A), TAGLN (transgelin; Module B), PLAU (urokinase; Module C), and SLMAP (sarcolemmal membrane-associated protein; Module D). In addition, Figure 3I depicts the pattern of the seed gene expression of all 11 modules. Regarding the nodes ≥ 10 and edges ≥ 10 in the modules, we subsequently used FUNRICH software for the functional analysis of the top four modules (Figure 3E‒H). According to the percentages of genes involved in progression, the results described the top 10 potential biological pathways of the DEGs. The findings indicated that the significant modules most enriched in the “cell cycle mitotic” (62% of the DEGs; Figure 3E), “smooth muscle contraction” (78.6%; Figure 3F), “proteoglycan syndecan-mediated signaling events” (85.7%; Figure 3G) and “ErbB receptor signaling network” (85.7%; Figure 3G), and “potassium channels” (50%; Figure 3H).

### 3.4. Hub Gene Selection and Analysis

**Figure 4:**
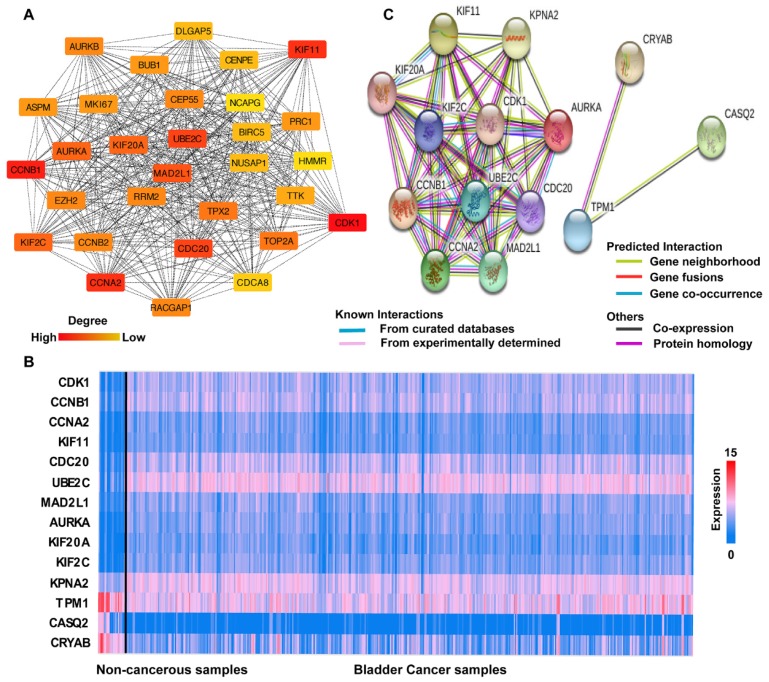
Interaction network analysis and expression of the hub genes. (**A**) The interactions and protein‒protein networks of the top 30 hub genes. (**B**) Hierarchical clustering of 14 hub genes analyzed in the present research. The samples on the left of the black bar are noncancerous bladder tissues from the TCGA-BLCA dataset (*n* = 19) and the samples on the right of the black bar are BCa samples (*n* = 406): high expression (red), low expression (blue). (**C**) Protein‒protein network and interaction among the 14 hub genes from STRING-db.org, accessed on 11 November 2019. Nodes with different colors represent different query proteins. A different color on the edge means a different interaction (see legend in figure).

Based on the findings of STRING analysis, the cytoHubba plugin in Cytoscape reported 376 hub genes of BCa in total, and 135 hub genes with the criterion of degree ≥11 connected nodes. The top 30 hub genes were selected, and the PPI network showed 30 nodes, 408 edges, and an average local clustering coefficient of 0.996 (Figure 4A). For deeper analysis, we focused on the top 10 hub genes, all showing a degree of ≥80. In addition, we included four genes (KPNA2, TPM1, CASQ2, and CRYAB) that were significantly correlated to overall survival, i.e., indicative of the prognosis of BCa (details shown in Section 3.5). Unfortunately, data on cancer-specific survival are not available in the TCGA-BLCA dataset.

The mutation rates of the 14 hub genes included in our current study were: CDK1(6/406, 1.5%); CCNB1 (9/406, 2.2%); CCNA2 (6/406, 1.5%); KIF11 (9/406, 2.2%); CDC20 (17/406, 4%); UBE2C (9/406, 2.2%); MAD2L1 (6/406, 1.5%); AURKA (4/406, 1%); KIF20A (7/406, 1.7%); KIF2C (17/406, 4%); KPNA2 (13/406, 3%); TPM1 (6/406, 1.5%); CASQ2 (11/406, 2.7%); and CRYAB (2/406,0.5%). The expression levels of the 14 hub genes in the noncancerous control and BCa specimens are shown in Figure 4B.

We then analyzed the interactions between the 14 hub genes in BCa samples by STRING online (Figure 4C). According to the counts in the gene set and *p*-value < 0.05, the top 10 categories of GO analysis and KEGG analysis for the hub genes were collected. The GO analysis described the BP mainly enriched in “cell division” and “mitotic cell cycle process”, the CC (cellular component) dominated in “spindle and cytosol”, while MF (molecular function) was mainly enriched in “enzyme binding”, “ATP binding”, and “protein kinase binding.” Furthermore, the KEGG was enriched in “oocyte meiosis”, “progesterone-mediated oocyte maturation”, “viral carcinogenesis”, and “cell cycle” (Table S5). The detailed information of names, abbreviations, and gene functions of the 14 hub genes are described in Table 2.

### 3.5. Clinical Analysis and Oncomine Analysis Outcomes of Hub Genes

**Figure 5:**
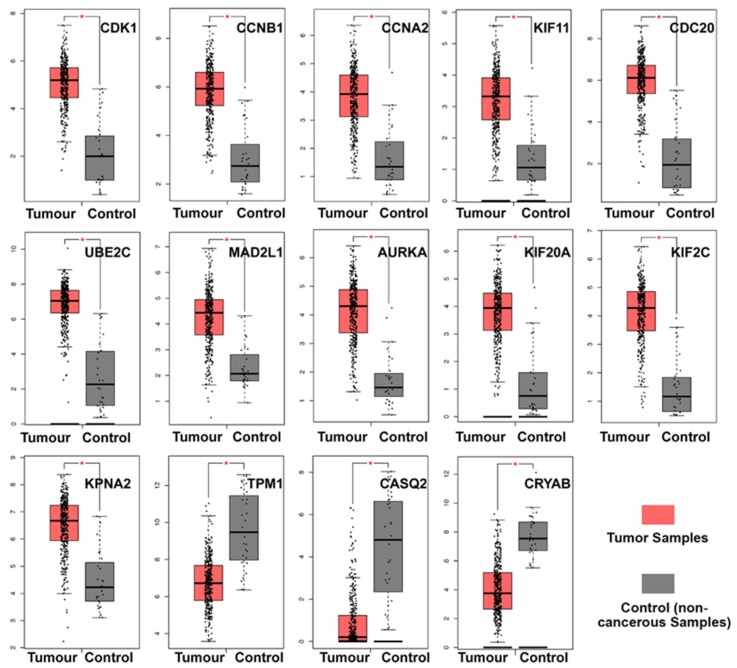
Box plots of gene expression levels for hub genes in noncancerous bladder samples and BCa samples. Y-axis = Log2 (TPM + 1). Control means the noncancerous samples from TCGA-BLCA (*n* = 19) and GTEx (*n* = 9). Tumor tissue, marked in red (*n* = 404), is also originally from TCGA-BLCA, with noncancerous tissues marked in gray (*n* = 28), and a red * indicating a *p*-value < 0.01. TPM = transcripts per million.

Compared with noncancerous bladder tissues, TPM1, CRYAB, and CASQ2 were significantly downregulated in BCa samples, while the other 11 hub genes were significantly upregulated (Figure 5). To elucidate the relevance of the hub genes in respect to tumor progression, we performed a multivariate analysis (ANOVA) of the BCa subgroups. The analysis included stages II‒IV [2] bladder cancer cases; stage I was omitted due to only two cases being available. TPM1 and CRYAB expression was significantly higher in stages III and IV, while CASQ2 expression was lower in stage II compared to stages III and IV. The other 11 hub genes showed no differential expression (Figure S2).

**Figure 6:**
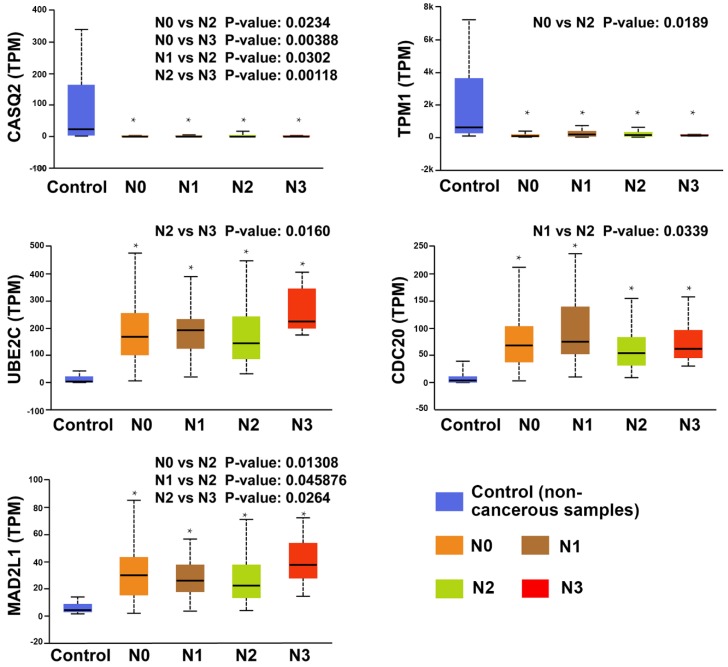
Box plots of gene expression levels of significantly altered hub genes in noncancerous bladder samples and BCa samples with different lymph nodal metastasis status. Controls = noncancerous samples from TCGA-BLCA (*n* = 19); Subgroups of BCa: N0 = no regional lymph node metastasis (*n* = 237 cases), N1 = metastases in 1–3 axillary lymph nodes (*n* = 46 cases), N2 = metastases in 4–9 axillary lymph nodes (*n* = 75 cases), and N3 = metastases in 10 or more axillary lymph nodes (eight cases) [23]. TPM = transcripts per million; * = significant difference compared with noncancerous tissues (*p* < 0.01).

Intriguingly, comparing the expression based on lymph nodal metastasis status (ANOVA) revealed significant downregulation of TPM1 and CASQ2 in all tumor groups compared to noncancerous bladder tissues, while no differences were found in the subgroups for CRYAB. The other 11 hub genes in all tumor groups were significantly upregulated compared to the control (*p* < 0.01). Furthermore, UBE2C, CDC20, MAD2L1, TPM1, and CASQ2 showed significant differences in the subgroups (Figure 6).

To investigate the possible clinical relevance of the identified hub genes, we used Kaplan–Meier survival analysis of overall survival (OS) and of disease-free survival (DFS). We first analyzed the top 10 hub genes with the highest expression levels (FPKM) and found that CCNB1 was correlated with a worse OS. Then, we tested all the hub genes with a degree ≥11 according to the cytoHubba analysis in Cytoscape. We found that the patients with low expression levels of four hub genes KPNA2 (degree 69), TPM1 (degree 29), CASQ2 (degree 11), and CRYAB (degree 11) showed better overall survival than patients with high expression did. Nevertheless, the OS analysis showed a significant difference in Rock2, ABCBCC9, CSRP1, NLAM1, CALD1, TUBA1A, and DLGA5. However, since an analysis of the data from the human protein atlas (https://www.proteinatlas.org/; accessed on 11 November 2019) revealed that KPNA2, TPM1, CASQ2, and CRYAB might be prognostic markers of BCa, we included those in our further analysis. 

**Figure 7:**
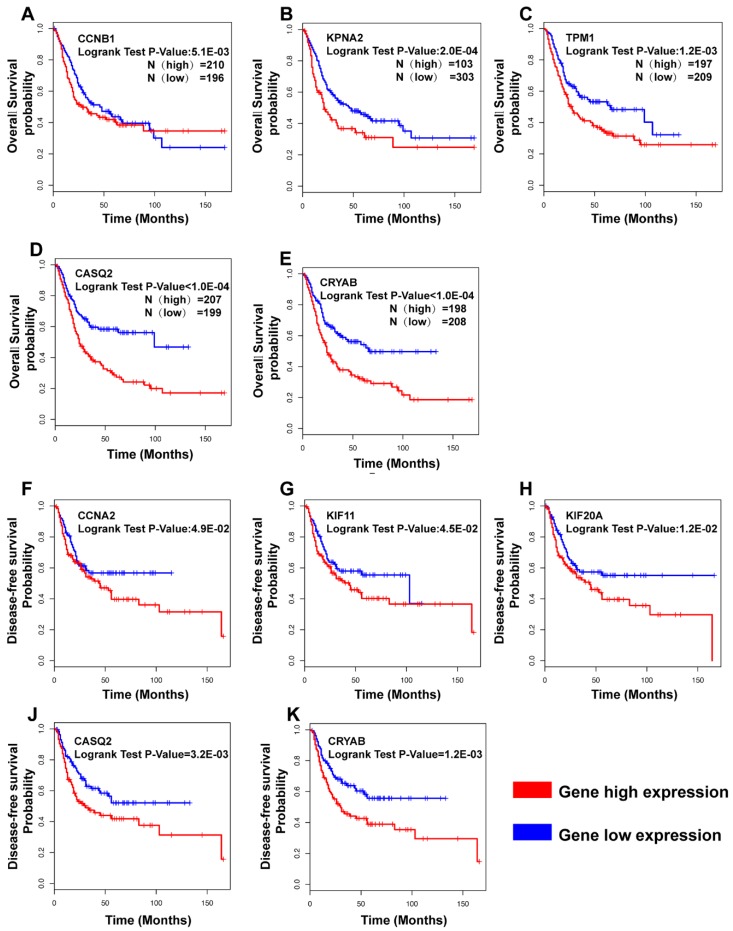
Overall survival and disease-free survival analyses of hub genes. Regarding the 14 hub genes analyzed, (**A**–**E**) show the significant results of OS; (**F**–**K**) show the significant results of DFS analysis. In DFS analysis, the cutoff—high and the cutoff—low each account for 50%.

Furthermore, the cases with low expression of CCNA2, KIF11, KIF20A, CASQ2, and CRYAB had a longer disease-free survival than the cases with high expression. In summary, based on the expression level, CCNB1, KPNA2, TPM1, CASQ2, and CRYAB were correlated with the overall survival, and CCNA2, KIF11, KIF2C, CASQ2, and CRYAB were correlated with the DFS (Figure 7).

**Figure 8:**
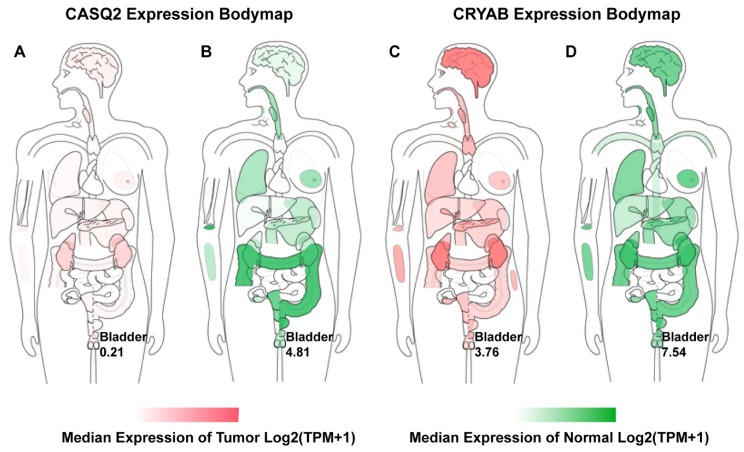
The median expression of CASQ2 and CRYAB in tumors (red) and normal tissues (green) in a body map. Deeper color represents higher expression levels. The map is based on the GEPIA database (http://gepia.cancer-pku.cn). TPM = transcripts per million.

With GEPIA (http://gepia.cancer-pku.cn, accessed on 11 November 2019) using data from the TCGA dataset, we contracted the expression body maps of CASQ2 and CRYAB. Both CASQ2 and CRYAB expression were lower in the bladders of BCa patients (Figure 8A,C). While CASQ2 was mainly downregulated in other organs except for the kidneys, CRYAB expression remained moderately high in all organs reviewed (Figure 8).

**Figure 9:**
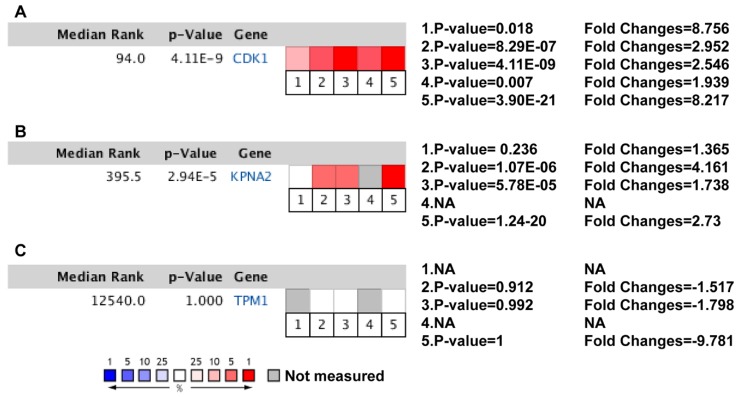
Oncomine meta-analysis of (**A**) CDK1, (**B**) KPNA2, and (**C**) TPM1 expression levels in BCa vs. no-cancerous bladder tissue. Heat maps of CDK1, KPNA2, and TPM1 gene expression in clinical BCa samples vs. noncancerous tissues. Clinical BCa samples are infiltrating bladder urothelial carcinoma. Data included from (1) Blaveri et al., *Clin Cancer Res*, 2005, invasive cancer samples *n* = 51, normal bladder samples *n* = 3 [33]. (2) Dyrskjot et al., *Cancer Res*, 2004, invasive cancer samples *n* = 13, normal bladder samples *n* = 14 [34]. (3) Lee et al., *J Clin Oncol*, 2010, invasive cancer samples *n* = 62, normal bladder samples *n* = 10 [35]. (4) Modlich et al., *Clin Cancer Res*, 2004, invasive cancer samples *n* = 20, normal bladder samples *n* = 4 [36]. (5) Sanchez-Carbayoet al., *J Clin Oncol*, 2006, invasive cancer samples *n* = 72, normal bladder samples *n* = 52 [37].

Finally, we performed an Oncomine analysis to analyze the expression of the representative hub genes in different datasets. Therefore, according to the degree, CDK1 is at the top of the hub genes, and, excluding enzyme binding, CDK1 has a role in the progression of the top categories of BP, CC, MF, and KEGG_Pathway collected in the present study. Regarding the OS and DFS results of hub genes, and based on the results of the Human Protein Atlas (HPA) (https://www.proteinatlas.org/; accessed on 11 November 2019), KPNA2 was upregulated and TPM1 was downregulated in BCa. Both are considered prognostic markers. Therefore, we selected CDK1, KPNA2, and TPM1 as representative genes for Oncomine meta-analysis. This analysis revealed significant upregulation of CDK1 and KPNA2 in BCa in previous major studies, while TPM1 expression was not altered (Figure 9).

## 4. Discussion

### 4.1. Biological Progression Potentially Associated with BCa

Our database analysis revealed 418 differentially expressed genes (DEGs) in BCa compared to noncancerous bladder tissue. Of those, we identified 14 hub genes for further analysis, which were enriched in cell cycle, mitotic cell cycle, and cytokinesis in a GO analysis. The pathways included pathways in cancer, cell cycle, viral carcinogenesis, MAPK signaling, and p53 signaling pathway. Biological pathways of four significant modules from PPI network analysis using Cytoscape contained “cell cycle, mitotic”, “epithelial to mesenchymal transition” (EMT), “ErbB receptor signaling” and “neuronal system” (Figure 3). Of note, “cell cycle” and “p53 signaling pathways” directly modulate the cell cycle in many cancers, and are an inevitable theme in cancer research [38,39]. Tumor suppressor p53 is the most frequently mutated gene in human cancer [40]. In BCa, the p53 expression also significantly declined, and was altered in the majority of aggressive BCa [41]. Interestingly, besides those directly cancer-related terms, “viral carcinogenesis” also showed up in a KEGG analysis. That is intriguing, since infection with polyomavirus BK could induce PCa in normal prostate epithelium (RWPE-1), but was not needed for maintaining the phenotype of PC3 prostate cancer cells in vitro [42].

We also found 12 downregulated genes related to the cGMP-PKG signaling pathway, a family of serine‒threonine kinases that contribute to the survival, growth, and metastatic potential of cancer cells. Downregulation of the cGMP-PKG is involved in maintaining PCa stemness, while pharmacological upregulation could prevent initiation, metastasis, and relapse of PCa [43]. Thus, cGMP-PKG signaling pathways may provide valuable targets for anti-PCa therapy.

EMT is the process by which tumor cells transit from adherent epithelial to mobile mesenchymal states, which facilitates cancer cells’ dissemination. Therefore, EMT plays an essential role in cancer metastasis and is highly relevant for the prognosis of cancer [44]. Moreover, EMT is also involved in BCa [45]. In BCa, Erben et al. showed that overexpression of ErbB2 is associated with an unfavorable prognosis. However, it is not an independent predictor of BCa [46]. The ErbB/MAPK signaling cascade might play an important role in BCa, and, at least in canine BCa models, Cronise et al. indicated that combined inhibition of MAPK and ErbB is probably an effective therapy [47]. 

### 4.2. The Hub Genes Are Potentially Associated with the Clinical Outcomes

Previous bioinformatics studies in BCa were either based on only one dataset [48,49,50], or the analyses used DNA sequencing [51], circular RNA [52], or DNA methylation [53]. Nevertheless, the recent study of Gao et.al analyzed four GEO databases [54], while the present study collected more public datasets to analyze. Here, we concentrated on mRNA expression of DEGs in noncancerous bladder tissues and BCa samples. The present study elucidates high-degree hub genes: CDK1, CCNB1, CCNA2, KIF11, CDC20, UBE2C, MAD2L1, AURKA, KIF20A, KIF2C, and KPNA2 had significant overexpression in BCa; TPM1, CASQ2, and CRYAB were significantly downregulated in BCa samples compared to noncancerous bladder tissues. In addition, some of the hub genes were in line with previous research (Table 3).

Our analysis also revealed that the expression of TPM1 and CRYAB was significantly higher in muscle invasive bladder cancer (MIBC) stages III and IV, and CASQ2 expression was higher in stage IV compared to stage II. This could indicate the association of TPM1, CRYAB, and CASQ2 with tumor progression. The other hub genes showed no significant association with tumor stage. Interestingly, based on lymph nodal metastasis status, all hub genes were significantly different from noncancerous tissue. This could indicate a role of the hub genes and related networks in BCa metastasis. However, we need further studies to elucidate whether those DEGs play a pivotal role in the lymph nodal metastasis of BCa.

In general, if target genes with high expression in cancer with a better prognosis are generally tumor suppressors, low expression with a better prognosis indicates an oncogene. CCNA2, CCNB1, KIF11, KIF20A, and KPNA2 are not only highly expressed in BCa samples, but also their high expression correlates with poor prognosis or poor DFS, which indicates that they are potential oncogenes. On the other hand, TPM1, CASQ2, and CRYAB have low expression in BCa samples, which may indicate that they could serve as tumor suppressors. In contrast, high expression of TPM1, CASQ2, and CRYAB was correlated with poor survival, while high expression of CASA2 and CRYAB was correlated with poor DFS. In a recent study, Liu et al. showed that upregulation of TPM1 and miR-96 by lncRNA MEG3 in bladder cancer cells inhibited cell proliferation, induced apoptosis in vitro, and inhibited tumor growth in a xenograft mouse model, supporting our idea of TPM1 as tumor suppressor [55]. Additionally, Thorsen et al. revealed that the mRNA expression level and protein expression level of TPM1 were downregulated in BCa [56].

Our finding from the TCGA database analysis of high TPM1 levels correlating with poor overall survival is in contrast to this idea. Of note, CDK1 and KPNA2 were significantly upregulated in the Oncomine meta-analysis and thus were in line with our TCGA-BLCA analysis, while TPM1 was unaltered in the meta-analysis but significantly downregulated in our analysis (Figure 5). CASQ2 seems to play a role in catecholaminergic polymorphic ventricular tachycardia research [57]. CRYAB was involved in a variety of signaling pathways implicated in breast cancer, lung cancer, prostate cancer, and ovarian cancer [58]. However, neither CASQ2 nor CRYAB has been studied in BCa yet. Those findings indicated that TPM1, CASQ2, and CRYAB signals need considerable and in-depth research.

### 4.3. The Hub Genes Potentially Associated with the Clinical Prognosis in Previous Research

Tian et al. demonstrated that CDK1 overexpression facilitated proliferation, migration, and invasion in BCa [59]. In addition, Shi et al. considered CDK1 and MAD2L1 crucial for the progression of lung cancer, since patients with increased CDK1 or MAD2L1 expression had a high risk of recurrence and poor prognosis [60]. However, few studies have reported a relationship between MAD2L1 and BCa; Choi reported that MAD2 and CDC20 were overexpressed in BCa, and closely correlated with high grade, advanced stage, and worsened OS in BCa [61]. 

CCNB1, a regulatory protein that plays an essential role in controlling mitosis, was upregulated in HCC patients with poor outcome [62,63]. Liu et al. reported that CCNB1 was closely correlated with GTSE1: after GTSE1 knockdown, CCNB1 and FoxM1 were significantly downregulated, while p53 expression was significantly increased in BCa. Furthermore, CCNB1 potentially contributed to tumor cell proliferation and migration and was positively correlated to disease recurrence and poor prognosis in BCa [64]. Kim highlighted the role of CCNB1 in predicting the risk of recurrence in non-muscle-invasive bladder cancer (NMIBC) [65]. CCNA2 was reported to be highly expressed in BCa with poor prognosis, and considered crucial for the progression of tumors [66]. Furthermore, Li indicated that CCNA2 induced the EMT progression through the ROCK/AKT/β-catenin/SNAIL pathway, and, in vitro, downregulated CCNA2 significantly repressed the proliferation and migration of BCa [67].

The Kinesin family members are involved in various physiological functions, like substance transport intracellular spindle formation and chromosome partitioning. Recent studies highlighted the role of KIF2C as an oncogene, and correlated it with the prognosis of lung cancer [68,69]. KIF11 is overexpressed in human cancers, including breast, lung, ovarian, and pancreatic cancer [70,71]. Nevertheless, until now, there have only been a few reports about KIF11 [72] and KIF2C [66] in BCa. Recently, Shen et al. observed that overexpression of KIF20A in BCa is associated with poor prognosis and worse tumor differentiation [73]. 

CDC20 serves as a critical protein in the spindle assembly checkpoint, cell cycle progression, and mitosis [74]. Kidokoro et al. demonstrated that the p53 pathway indirectly induced the expression of CDC20 in tumors [75]. Gayyed et al. analyzed CDC20 in BCa samples and normal tissues, with their results showing that CDC20 was negatively stained in normal tissues from the bladder, but was positive in BCa samples. Even more, high expression of CDC20 was associated with high tumor grade [76]. 

UBE2C is involved in the regulation of mitotic cyclins, cell cycle progression, and cancer progression [77]. Kim et al. demonstrated that UBE2C in the urine of BCa patients is significantly higher than in normal urine, and suggested it as a prognostic biomarker for BCa [78].

AURKA plays a crucial role in various mitotic events during chromosome segregation. As a target gene of β-catenin, it is involved in many cancers, such as gastric cancer, HCC, and esophageal squamous cell carcinoma [79,80]. Guo et al. reported that AURKA overexpression in BCa was strongly associated with tumor stage and grade; additionally, AURKA upregulation was correlated with poor overall survival [81]. In xenograft BCa models, the therapeutic potential of AURKA blockade was demonstrated [82]. 

KPNA2 is overexpressed in BCa and associated with poor prognosis [83]. Moreover, the expression levels of KPNA2 and OCT4 are significantly associated with primary tumor stage [84].

### 4.4. Major Findings of the Recent Study

In summary, the present study identified 418 DEGs and 376 hub genes. In comparison with noncancerous bladder tissues, 14 genes were studied in detail: CDK1, CCNB1, CCNA2, KIF11, CDC20, UBE2C, MAD2L1, AURKA, KIF20A, KIF2C, and KPNA2. However, a literature search revealed that the hub genes we found have not been widely reported in BCa, especially MAD2L1, KIF11, KIF2C, CSAQ2, CRYAB, TPM1, and KPNA2. The most interesting findings were:

(i) TPM1, CRYAB, and CASQ2 were significantly downregulated in BCa, while the remaining 11 hub genes were significantly upregulated.
(ii) All hub genes showed a significant difference between the lymph node metastasis status and noncancerous tissues. In particular, UBE2C, CDC20, MAD2L1, TPM1, and CASQ2 potentially play a pivotal role in lymph node metastasis.
(iii) CCNB1, KPNA2, TPM1, CASQ2, and CRYAB were correlated with prognosis in overall survival (OS) analysis, while CCNA2, KIF11, KIF20A, CASQ2, and CRYAB were correlated with disease-free survival (DFS). Interestingly, CRYAB and CASQ2 were correlated with both OS and DFS.
(iv) We here report evidence for an up to now unrecognized possible role of CASQ2 in cancer and for CRYAB in bladder cancer. In combination with the results from the HPA, our study suggests that CRYAB would be a candidate biomarker in BCa, followed by CASQ2, TPM1, and KPNA2.

## 5. Conclusions

In conclusion, the bioinformatics analysis of differentially expressed genes in bladder cancer, using mRNA expression data from several public databases, revealed several candidate genes that may be directly or indirectly involved in BCa carcinogenesis, progression, or metastasis according to a literature survey. All of the above suggests that the hub genes represent potential core molecular pathways involved in the carcinogenesis, progression, and recurrence of BCa, promoting our understanding of the mechanisms and progression of BCa, and indicating that these hub genes are potential candidate biomarkers for BCa. However, our in silico study is limited and needs validation of the cancer biological functions of the potential biomarkers in future patient studies. Meanwhile, ongoing cell culture and tissue studies in our laboratory seek to confirm these results.

## Figures and Tables

**Table 1:** General information on GEO datasets and platforms.

Dataset	Number of Noncancerous Bladder Tissue Samples	Number of Cancer Tissue Samples	Number of DEGs	Platform	Manufacturer of Platform
GSE27448	5	10	4701	GPL2895	GE Healthcare
GSE52519	3	9	742	GPL16884	JPT Peptide Technology
GSE61615	2	2	736	GPL14550	Agilent Technologies
GSE76211	3	3	658	GPL17586	Affymetrix
GSE100926	3	3	194	GPL16956	Agilent Technologies

**Table 2:** Functional roles and general information of the 14 hub genes in BCa.

Gene Symbol	Other Names	Full Name	Role of Genes	Putative or Observed Effect
CDK1	CDC2, CDC28A, P34CDC2	Cyclin-dependent kinase 1	Oncogene	Overexpression in BCa; Activating CDK1 facilitates the proliferation and invasion of BCa. [23]
CCNB1	cyclin B1	CCNB	Oncogene	Increased expression in BCa; Regulating BCa through the FoxM1/CCNB1 pathway, correlates with poor prognosis. [24]
KIF11	kinesin family member 11	EG5; HKSP; KNSL1; MCLMR; TRIP5	Oncogene	Overexpression in BCa; Predicted to be Oncogene. [25]
CCNA2	cyclin A2	CCN1; CCNA	Oncogene	Over expression in BCa; Activity of CCNA2 regulated the EMT via ROCK/AKT/β-catenin/SNAIL pathway to influence the prognosis of BCa. [26]
UBE2C	ubiquitin conjugating enzyme E2 C	UBCH10; dJ447F3.2	Oncogene	High expression in BCa; Activating UBE2C induced progression and correlates with poor survival. [27,28]
CDC20	cell division cycle 20	CDC20A; p55CDC; bA276H19.3	Oncogene	Increased expression in BCa; Activating CDC20 increased carcinogenesis and correlates with poor survival in BCa. [29]
MAD2L1	mitotic arrest deficient 2 like 1	MAD2; HSMAD2	Oncogene	Overexpression in BCa; Activating MAD2 was associated with incidence of recurrence and progression of BCa. [30]
KIF2C	kinesin family member 2C	MCAK; CT139; KNSL6	Oncogene	Overexpression of KIF2C was confirmed in BCa from rat model. [31]
AURKA	aurora kinase A	AIK; ARK1; AURA; BTAK; STK6; STK7; STK15; PPP1R47	Oncogene	Highly expression in BCa; Activating AURKA is associated with poor prognosis, tumor stage and grade. [32,33]
KIF20A	kinesin family member 20A	MKLP2; RAB6KIFL	Oncogene	Over expression in BCa; KIF20A was associated with the development of BCa and correlated with poor survival. [34]
KPNA2	karyopherin subunit alpha 2	QIP2; RCH1; IPOA1; SRP1alpha; SRP1-alpha	Oncogene	Increased expression in BCa; Activation of KPNA2 defines poor prognosis in BCa. [35,36]
TPM1	tropomyosin 1	CMH3; TMSA; CMD1Y; LVNC9; C15orf13;	Tumor suppressor	Downregulated in BCa; Activating TPM1 inhibited the proliferation and promoted the apoptosis of Bladder tumor cells. [37,38]
CASQ2	calsequestrin 2	PDIB2	Disease-related gene	Reduced in BCa in present study; Predicted to be tumor suppressor; No study of CASQ2 reported in BCa. [39]
CRYAB	crystallin alpha B	MFM2; CRYA2; CTPP2; HSPB5; CMD1II; CTRCT16;	Cancer-related gene	Decreased expression in BCa in present study; Enforced activation of CRYAB correlated to poor prognosis of lung cancer and other tumors [40]; No study of CRYAB reported in BCa.

**Table 3:** Hub genes predicted and studied in previous bioinformatics analyses of BCa.

Hub Genes			Reference
Upregulated	Downregulated	Regulation not Specified	(Databases Used)
AURKA *, CCNA2 *, CCNE1#, CDC20 *, BCL3#, CEP55 *, DCUN1D1#, FGFR1OP#, **HMMR** *, MAP3K8#, MYB#, PTG1#, VEGFA#	DUSP26#, MEIS1 *		Han et al. [48] (GSE52519)
CDC20 *	ACTA#, DCN#, MYH11 *, TPM1 *, VIM#, TGFB3#		Hu et al. [49] (GSE13507, TCGA-BLCA)
		ITGA7#, GRB14#, CDC20 *, PSMB1#, POLR2F/2H#, RPS14/15#	Jia et al. [50] (GSE27448)
		EME1#, AKAP9#, ZNF91#, OARD3#, STAG2#, ZFP36L2#, METTL3#, POLR3#, MUC7#	Han et al. [51] (TCGA-BLCA)
DSN1#, KNTC1 *, CDK1 *, CENPM#, HIST1H2BJ#, EZH2 *, CENPF *, RAD51#, BRCA1#, EXO1#			Jiang and Yuan [52] (Circular RNA Interactome database; TCGA)
CDH1#, DDOST#, CASP8#, DHX15#, PTRF@	GNG4#, ADCY9#, NPY#, ADRA2B#, PENK#		Zhang et al. [53] (GSE3167, GSE65635, GSE33510)
TOP2A *, CDC20 *, UBE2C *, CCNB1 *, CCNB2 *, VEGFA#, AURKB *, AURKA *, CEP55 *	ACTA2#		Gao et al. [54] (GSE7476, GSE13507, GSE37815, GSE65635)

(*) consistent with the present study (bold face); (@) opposite to the present study; (#) not defined as hub genes in the present study.

## Supplementary Materials

The following are available online at https://www.mdpi.com/2075-4418/10/2/66/s1, Figure S1: Quality analysis, Figure S2: Expression level for hub genes in the different BCa stages, Table S1: GOTERM_BP analysis, Table S2: GOTERM_CC analysis, Table S3: GOTERM_MF; Table S4: KEGG analysis, Table S5: GO and KEGG analysis of hub genes.

## Abbreviations

BCa	Bladder cancer
GEO	Gene Expression Omnibus
TCGA-BLCA	Cancer Genome Atlas Bladder Cancer
GO	Gene ontology
KEGG	Kyoto Encyclopedia of Genes and Genomes
PPI	Protein‒protein interaction
FC	Fold change
RLE	Relative log expression
OS	Overall survival
DFS	Disease-free survival
HJURP	Holliday junction recognition protein
TAGLN	Transgelin
PLAU	Urokinase
SLMAP	Sarcolemmal membrane-associated protein
NMIBC	Non-muscle-invasive bladder cancer
MIBC	Muscle-invasive bladder cancer
DEGs	Differentially expressed genes
MCODE	Molecular complex detection
GEPIA	Gene expression profiling interactive analysis
BP	Biological process
CC	Cellular component
MF	Molecular function
cGMP-PKG	Cyclic guanosine monophosphate-dependent protein kinase
cAMP	Cyclic adenosine monophosphate
FPKM	Fragments per kilobase million
TPM	Transcripts per million
