# Supplementary material for: Identification of Key Biomarkers in Bladder Cancer: Evidence from a Bioinformatics Analysis
Source: Diagnostics (Basel). 2020 Jan 24;10(2):66. doi: 10.3390/diagnostics10020066 (PMC7168923; doi:10.3390/diagnostics10020066)

Figure S1：Quality analysis. Box plots of GSE datasets before and after normalization. The medians almost present a straight line indicating high data quality.

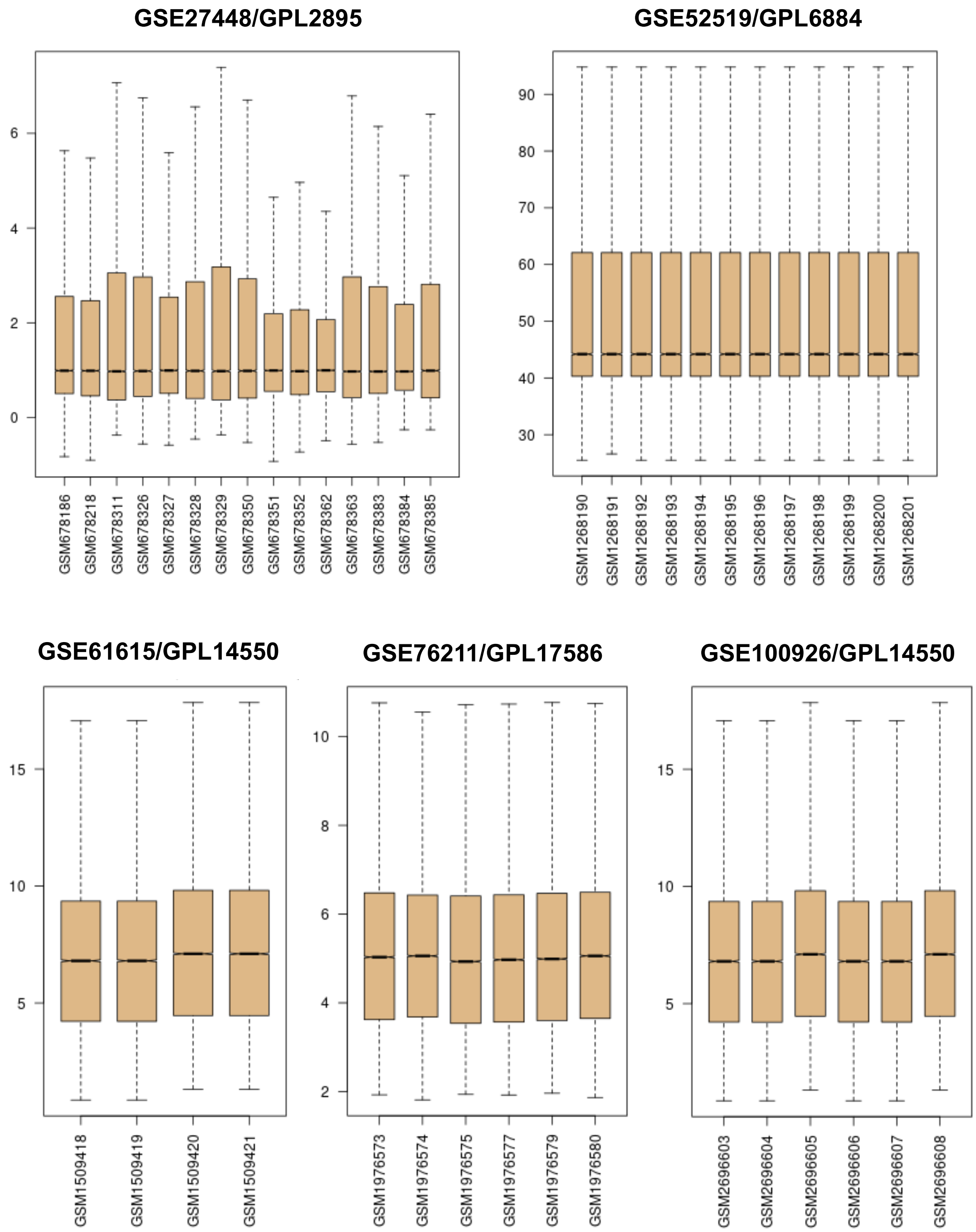

Supplement: Supplementary file 1 [file diagnostics-10-00066-s001.zip › Figure S1.pdf]
