# Supplementary material for: Identification of Key Biomarkers in Bladder Cancer: Evidence from a Bioinformatics Analysis
Source: Diagnostics (Basel). 2020 Jan 24;10(2):66. doi: 10.3390/diagnostics10020066 (PMC7168923; doi:10.3390/diagnostics10020066)
Supplement: Supplementary file 1 [file diagnostics-10-00066-s001.zip › Table S1.pdf]

Table S1. GOTERM_BP analysis. Analysis of total DEGs and the subgroups of up- and down-regulated DEGs. Numbers in brackets indicate count of DEGs.

ID	Description	Count in Gene set	Percent -age%	P-value	Benjamini	List of Genes	
						**Up-regulated**	**Down-regulated**
**Analysis of Total DGEs**							
**GO:0051301**	**Cell division**	**61**	**14.6**	**5.5E-23**	**2.8E-19**	PRC1 ASPM AURKA AURKB BIRC5 BLM CCNA2 CCNB2 CDC20 CDC25C CDCA5 CDCA8 CDK1 CENPE CENPF CEP55 CIT CKAP2 CKS1B CKS2 DIXDC1 ECT2 FGF2 FZD7 KDF1 KIF11 KIF20A KIF2C KLHL42 KNTC1 MAD2L1 NCAPD2 NCAPG NEK2 NUSAP1 PTCH1 PTTG1 RACGAP1 ROCK2 RUVBL1 SMC4 TACC1 TACC3 TOP2A TPX2 TUBB TXNIP UBE2C UBE2S ZBTB16 ZWINT*(51)*	BUB1 CCNB1 FGF9 KIF13A MCM5 RHOB STMN1 SVIL TUBA1A TUBA1C *(10)*
**GO:1903047**	**Mitotic cell cycle process**	**74**	**17.7**	**1.3E-21**	**3.2E-18**	STIL PRC1 EZH2 KNTC1 TTK AURKA PTTG1 AURKB MCM10 KIF2C CDCA8 FANCI RANBP1 TOP2A CDCA5 CCNA2 ASPM CDK1 KIF11 KIF15 TPX2 NUSAP1 MCM2 UBE2C TACC3 MCM5 NCAPD2 INHBA MAD2L1 RRM2 ZWINT STMN1 RUVBL1 UBE2S MELK CKS1B BLM NEK2 FOXM1 CHEK1 CEP55 HMMR TYMS TUBB NCAPG BUB1 TFDP1 CKAP2 DLGAP5 CENPF CENPE CDC20 BIRC5 CDC25C RACGAP1 CDKN3 SMC4 CCNB1 PLK4 CCNB2 BAX PCNACKS2 CIT KIF20A *(65)*	FGF10 TUBA1A PID1 FLNA FHL1 CAMK2G PKD2 KLHL42 ID4 *(9)*
**GO:0000278**	**Mitotic cell cycle**	**76**	**18.2**	**9.3E-21**	**1.6E-17**	STIL PRC1 EZH2 KNTC1 TTK AURKA PTTG1 AURKB MCM10 PAK6 KIF2C CDCA8 FANCI RANBP1 TOP2 CDCA5 CCNA2 ASPM CDK1 KIF11 KIF15 TPX2 NUSAP1 MCM2 UBE2C TACC3 MCM5 NCAPD2 INHBA MAD2L1 RRM2 ZWINT STMN1 RUVBL1 UBE2S MELK CKS1B BLM NEK2 FOXM1 CHEK1 CEP55 HMMR TYMS TUBB NCAPG BUB1 TFDP1 CKAP2 MKI67 DLGAP5 CENPF CENPE CDC20 BIRC5 CDC25C RACGAP1 CDKN3 SMC4 CCNB1 PLK4 CCNB2 BAX PCNA CKS2 CIT KIF20A *(67)*	FGF10 TUBA1A PID1 FLNA FHL1 CAMK2G PKD2 KLHL42 ID4 *(9)*
**GO:0003012**	**Muscle system process**	**47**	**11.2**	**3.1E-19**	**4.0E-16**	EZH2 TPM3 SCO2 DSC2 *(4)*	PTGS2 CAMK2G CALD1 CACNB2 TPM2 TPM1 MYL9 VCL ANXA6 ACTG2 ATP2B4 DES SORBS1 SORBS2 DMD SLMAP CNN1 LMOD1 MYOM1 ERRFI1 DTNA RCSD1 KCNMA1 KCND3 ACTC1 PTGER3 ROCK2 CRYAB ATP1A2 SSPN FLNA HSPB6 CHRM3 PLN PDE5A MYH11 AKAP6 SYNM GAMT CASQ2 SGCA MYLK TMOD1 *(44)*
**GO:0022402**	**Cell cycle process**	**87**	**20.8**	**3.3E-18**	**3.4E-15**	STIL PRC1 EZH2 KNTC1 TTK AURKA AURKB PTTG1 MCM10 KIF2C CDCA8 FANCI FAP RANBP1 TOP2A CCNA CDCA5 ASPM DHCR24 CDK1 KIF11 KIF15 TPX2 NUSAP1 MCM2 UBE2C TACC3 ECT2 MCM5 NCAPD2 INHBA MAD2L1 RRM2 ZWINT STMN1 RUVBL1 UBE2S MELK CKS1B BLM NEK2 FOXM1 CHEK1 CEP55 HMMR TYMS TUBB NCAPG BUB1 FEN1 TFDP1 TRIP13 CKAP2 MKI67 DLGAP5 CENPF CENPE CDC20 BIRC5 CDC25C RACGAP1 CDKN3 SMC4 CCNB1 PLK4 CCNB2 BAX PCNA CKS2 CIT KIF20A *(71)*	FGF10 KIF13A ATP2B4 ILK RHOB TUBA1A PID1 ROCK2 FLNA FHL1 CAMK2G PKD2 KLHL42 C2ORF40 SVIL ID4 *(16)*
**GO:0044772**	**Mitotic cell cycle phase transition**	**51**	**12.2**	**5.7E-18**	**4.9E-15**	EZH2 KNTC1 TTK AURKA MCM10 FANCI RANBP1 CCNA2 CDCA5 CDK1 TPX2 MCM2 TACC3 UBE2C MCM5 INHBA MAD2L1 RRM2 UBE2S MELK CKS1B BLM NEK2 FOXM1 CHEK1 HMMR TYMS TUBB BUB1 TFDP1 DLGAP5 CENPF BIRC5 CDC20 CENPE CDKN3 CDC25C CCNB1 PLK4 CCNB2 BAX CKS2 PCNA CIT *(44)*	FGF10 TUBA1A PID1 FHL1 CAMK2G PKD2 ID4 *(7)*
**GO:0007049**	**Cell cycle**	**97**	**23.2**	**1.7E-17**	**1.1E-14**	STIL PRC1 DTYMK KNTC1 TTK AURKA PTTG1 AURKB MCM10 CDCA8 FAP CCNA2 CDCA5 ASPM TACC3 NCAPD2 MAD2L1 ZWINT STMN1 BLM NEK2 CHEK1 TUBB TFDP1 CKAP2 MKI67 CDC20 PLK4 PCNA E2F2 EZH2 PAK6 KIF2C FANCI RANBP1 TOP2A DHCR24 CDK1 KIF11 KIF15 TPX2 NUSAP1 MCM2 UBE2C ECT2 MCM5 INHBA RRM2 RUVBL1 UBE2S MELK CKS1B FOXM1 CEP55 HMMR TYMS HJURP NCAPG BUB1 FEN1 TRIP13 DLGAP5 CENPF CENPE BIRC5 CDKN3 RACGAP1 CDC25C SMC4 CCNB1 CCNB2 BAX CKS2 CIT KIF20A *(75)*	PTGS2 FGF10 KIF13A ATP2B4 ILK PID1 ROCK2 TACC1 HHEX DIXDC1 C2ORF40 RHOB TUBA1A FLNA CAMK2G FHL1 PKD2 KLHL42 TXNIP SVIL PTCH1 ID4 *(22)*
**GO:0044770**	**Cell cycle phase transition**	**52**	**12.4**	**1.6E-17**	**1.2E-14**	EZH2 KNTC1 TTK AURKA MCM10 FANCI RANBP1 CCNA2 CDCA5 CDK1 TPX2 MCM2 TACC3 UBE2C MCM5 INHBA MAD2L1 RRM2 UBE2S MELK CKS1B BLM NEK2 FOXM1 CHEK1 HMMR TYMS TUBB BUB1 TFDP1 DLGAP5 CENPF BIRC5 CDC20 CENPE CDKN3 CDC25C CCNB1 PLK4 CCNB2 BAX CKS2 PCNA CIT *(44)*	FGF10 ATP2B4 TUBA1A PID1 FHL1 CAMK2G PKD2 ID4 *(8)*
**GO:1902589**	**Single-organism organelle organization**	**92**	**22.0**	**6.4E-16**	**3.8E-13**	STIL PRC1 KNTC1 TTK AURKA AURKB KIF2C CDCA8 RANBP1 TOP2A CDCA5 ASPM CDK1 KIF11 TPX2 NUSAP1 UBE2C TACC3 NCAPD2 MAD2L1 ZWINT STMN1 ARL6IP1 NEK2 DIAPH3 KIAA0101 CHEK1 CEP55 TPM3 TUBB NCAPG BUB1 FEN1 TFDP1 CKAP2 DLGAP5 CENPF CENPE CDC20 BIRC5 RACGAP1 SMC4 CCNB1 PLK4 BAX PCNA CIT KIF20A *(48)*	RHOJ SNCA PDLIM3 FGF10 KANK1 DSTN DES ANG MAPT RAB23 LMOD1 PID1 STX2 CRYAB ROCK2 ACTN1 PALLD TACC1 FLNA STXBP6 JAM3 DST CASQ2 MAP7D3 TPM2 TPM1 SORBS1 SORBS2 DMD PKD2 CNN1 FYCO1 ACTC1 NTF3 MAP1A SYT11 MAP1B SYNPO2 NTRK3 SYNE1 MYH11 ABCC4 SYNM TMOD1 *(44)*
**GO:0007010**	**Cytoskeleton organization**	**74**	**17.7**	**1.9E-15**	**9.7E-13**	STIL PRC1 TTK AURKA AURKB PAK6 KIF2C RANBP1 ASPM TUBA1C CDK1 KIF11 TPX2 NUSAP1 TACC3 STMN1 NEK2 DIAPH3 KIAA0101 CHEK1 TPM3 TUBB CKAP2 BIRC5 RACGAP1 PLK4 CIT KIF20A *(28)*	RHOJ SNCA PDLIM3 FGF10 KANK1 DSTN DES ANG MAPT ILK TUBB6 LMOD1 TUBA1A CRYAB ROCK2 ACTN1 PALLD TACC1 FLNA ZNF135 JAM3 DST CASQ2 MAP7D3 TPM2 TPM1 SORBS1 SORBS2 DMD PKD2 CNN1 DIXDC1 ACTC1 NTF3 MAP1A MAP1B SYNPO2 EPHA3 NTRK3 SYNE1 SVIL MYH11 SYNM SH3D19 TMOD1 ARHGAP10 *(46)*
**Further cancer-related GOTERMS**							
**GO:0032970**	**Regulation of actin filament-based process**	**18**	**4.3**	**2.3E-3**	**4.1E-2**	DSC2 CIT *(2)*	DIXDC1 NTF3 ROCK2 SYNPO2 ATP1A2 TPM1 FLNA KANK1 DSTN EPHA3 NTRK3 PLN ILK LMOD1 JAM3 TMOD1 *(16)*
**GO:0000910**	**Cytokinesis**	**15**	**3.6**	**7.9E-7**	**8.6E-5**	CKAP2 PRC1 NUSAP1 BIRC5 AURKB CEP55 RACGAP1 ECT2 STMN1 CIT KIF20A *(11)*	ROCK2 KIF13A SVIL RHOB *(4)*
**GO:0048285**	**Organelle fission**	**47**	**11.2**	**3.9E-12**	**1.2E-9**	PRC1 NEK2 KNTC1 TTK CHEK1 AURKA CEP55 PTTG1 AURKB MTFR2 KIF2C CDCA8 NCAPG BUB1 RANBP1 TOP2A CDCA5 CCNA2 ASPM TRIP13 CDK1 KIF11 MKI67 DLGAP5 KIF15 TPX2 CENPF NUSAP1 CENPE CDC20 BIRC5 RACGAP1 UBE2C TACC3 CDC25C SMC4 NCAPD2 CCNB1 CCNB2	KLHL42 FLNA *(2)*

						MAD2L1 ZWINT CKS2 RUVBL1 CIT UBE2S *(45)*	
GO:0000280	Nuclear division	46	11.0	1.6E-12	5.3E-10	PRC1 NEK2 KNTC1 TTK CHEK1 AURKA CEP55 PTTG1 AURKB KIF2C CDCA8 NCAPG BUB1 RANBP1 TOP2A CDCA5 CCNA2 ASPM TRIP13 CDK1 KIF11 MKI67 DLGAP5 KIF15 TPX2 CENPF NUSAP1 CENPE CDC20 BIRC5 RACGAP1 UBE2C TACC3 CDC25C SMC4 NCAPD2 CCNB1 CCNB2 MAD2L1 ZWINT CKS2 RUVBL1 CIT UBE2S *(44)*	KLHL42 FLNA *(2)*
**Analysis of Up-regulated DGEs**							
GO:0007049	Cell cycle	75	56.8	1.40E-41	9.20E-39	STIL, E2F2, PRC1, EZH2, DTYMK, KNTC1, TTK, AURKA, PTTG1, AURKB, MCM10, PAK6, KIF2C, CDCA8, FANCI, FAP, RANBP1, TOP2A, CDCA5, CCNA2, ASPM, DHCR24, CDK1, KIF11, KIF15, TPX2, NUSAP1, MCM2, UBE2C, TACC3, ECT2, MCM5, NCAPD2, INHBA, MAD2L1, RRM2, ZWINT, STMN1, RUVBL1, UBE2S, MELK, CKS1B, BLM, NEK2, FOXM1, CHEK1, CEP55, HMMR, TYMS, TUBB, HJURP, NCAPG, BUB1, FEN1, TFDP1, TRIP13, CKAP2, MKI67, DLGAP5, CENPF, CENPE, CDC20, BIRC5, CDC25C, RACGAP1, CDKN3, SMC4, CCNB1, PLK4, CCNB2, BAX, PCNA, CKS2, CIT, KIF20A *(75)*	
GO:0022402	Cell cycle process	71	53.8	3.40E-43	3.00E-40	STIL, PRC1, EZH2, KNTC1, TTK, AURKA, PTTG1, AURKB, MCM10, KIF2C, CDCA8, FANCI, FAP, RANBP1, TOP2A, CDCA5, CCNA2, ASPM, DHCR24, CDK1, KIF11, KIF15, TPX2, NUSAP1, MCM2, UBE2C, TACC3, ECT2, MCM5, NCAPD2, INHBA, MAD2L1, ZWINT, RRM2, STMN1, RUVBL1, UBE2S, MELK, CKS1B, BLM, NEK2, FOXM1, CHEK1, CEP55, HMMR, TYMS, TUBB, NCAPG, BUB1, FEN1, TFDP1, TRIP13, CKAP2, MKI67, DLGAP5, CENPF, CENPE, CDC20, BIRC5, RACGAP1, CDKN3, CDC25C, SMC4, CCNB1, PLK4, CCNB2, BAX, PCNA, CKS2, CIT, KIF20A *(71)*	
GO:0000278	Mitotic cell cycle	67	50.8	6.10E-48	1.60E-44	STIL, PRC1, EZH2, KNTC1, TTK, AURKA, PTTG1, AURKB, MCM10, PAK6, KIF2C, CDCA8, FANCI, RANBP1, TOP2A, CDCA5, CCNA2, ASPM, CDK1, KIF11, KIF15, TPX2, NUSAP1, MCM2, TACC3, UBE2C, MCM5, NCAPD2, INHBA, MAD2L1, ZWINT, RRM2, STMN1, RUVBL1, UBE2S, MELK, CKS1B, BLM, NEK2, FOXM1, CHEK1, CEP55, HMMR, TYMS, TUBB, NCAPG, BUB1, TFDP1, CKAP2, MKI67, DLGAP5, CENPF, CENPE, CDC20, BIRC5, RACGAP1, CDKN3, CDC25C, SMC4, CCNB1, PLK4, CCNB2, BAX, PCNA, CKS2, CIT, KIF20A *(67)*	
GO:1903047	Mitotic cell cycle process	65	49.2	1.10E-47	1.40E-44	STIL, PRC1, EZH2, KNTC1, TTK, AURKA, PTTG1, AURKB, MCM10, KIF2C, CDCA8, FANCI, RANBP1, TOP2A, CDCA5, CCNA2, ASPM, CDK1, KIF11, KIF15, TPX2, NUSAP1, MCM2, TACC3, UBE2C, MCM5, NCAPD2, INHBA, MAD2L1, ZWINT, RRM2, STMN1, RUVBL1, UBE2S, MELK, CKS1B, BLM, NEK2, FOXM1, CHEK1, CEP55, HMMR, TYMS, TUBB, NCAPG, BUB1, TFDP1, CKAP2, DLGAP5, CENPF, CENPE, CDC20, BIRC5, RACGAP1, CDKN3, CDC25C, SMC4, CCNB1, PLK4, CCNB2, BAX, PCNA, CKS2, CIT, KIF20A *(65)*	
GO:0051726	Regulation of cell cycle	51	38.6	4.00E-29	9.30E-27	STIL, E2F2, PRR11, EZH2, KNTC1, TTK, AURKA, AURKB, FANCI, FAP, RANBP1, TOP2A, CDCA5, CCNA2, ASPM, DHCR24, CDK1, KIF11, TPX2, NUSAP1, TACC3, UBE2C, ECT2, INHBA, MAD2L1, ZWINT, CKS1B, BLM, NEK2, FOXM1, KIAA0101, CHEK1, BUB1, FEN1, TFDP1, MKI67, DLGAP5, CENPF, BIRC5, CDC20, CENPE, RACGAP1, CDKN3, CDC25C, CCNB1, PLK4, CCNB2, BAX, CKS2, PCNA, CIT *(51)*	
GO:1902589	Single-organism organelle organization	48	36.4	4.80E-17	5.70E-15	ARL6IP1, STIL, PRC1, NEK2, DIAPH3, KNTC1, KIAA0101, TTK, CHEK1, AURKA, CEP55, AURKB, TPM3, KIF2C, CDCA8, TUBB, NCAPG, BUB1, RANBP1, TOP2A, CDCA5, FEN1, ASPM, TFDP1, CKAP2, CDK1, KIF11, DLGAP5, TPX2, CENPF, NUSAP1, CENPE, CDC20, BIRC5, RACGAP1, TACC3, UBE2C, SMC4, NCAPD2, CCNB1, PLK4, MAD2L1, BAX, ZWINT, PCNA, STMN1, CIT, KIF20A *(48)*	
GO:0051301	Cell division	47	35.6	4.30E-35	2.20E-32	CKS1B, PRC1, BLM, NEK2, KNTC1, AURKA, CEP55, AURKB, PTTG1, KIF2C, CDCA8, TUBB, KDF1, NCAPG, BUB1, TOP2A, CDCA5, CCNA2, ASPM, TUBA1C, CKAP2, CDK1, KIF11, TPX2, CENPF, NUSAP1, CENPE, CDC20, BIRC5, RACGAP1, UBE2C, TACC3, CDC25C, ECT2, MCM5, SMC4, NCAPD2, CCNB1, CCNB2, MAD2L1, ZWINT, CKS2, STMN1, RUVBL1, CIT, UBE2S, KIF20A *(47)*	
GO:0006464	Cellular protein modification process	46	34.8	7.60E-04	1.10E-02	CKS1B, BLM, NEK2, MMP9, FOXM1, EZH2, TTK, CBX2, CHEK1, AURKA, PTTG1, AURKB, PAK6, CDCA8, FANCI, GRIN2D, MARVELD3, FUT3, BUB1, PPP1R14C, TOP2A, CCNA2, CDK1, SHMT2, DLGAP5, TPX2, CENPE, CDC20, BIRC5, UBE2C, CDKN3, CDC25C, ECT2, CCNB1, INHBA, PLK4, MAD2L1, BAX, PCNA, SNRPB, CKS2, RUVBL1, CIT, UBE2S, UBE2T, MELK	
GO:0036211	Protein modification process	46	34.8	7.60E-04	1.10E-02	CKS1B, BLM, NEK2, MMP9, FOXM1, EZH2, TTK, CBX2, CHEK1, AURKA, PTTG1, AURKB, PAK6, CDCA8, FANCI, GRIN2D, MARVELD3, FUT3, BUB1, PPP1R14C, TOP2A, CCNA2, CDK1, SHMT2, DLGAP5, TPX2, CENPE, CDC20, BIRC5, UBE2C, CDKN3, CDC25C, ECT2, CCNB1, INHBA, PLK4, MAD2L1, BAX, PCNA, SNRPB, CKS2, RUVBL1, CIT, UBE2S, UBE2T, MELK *(46)*	
GO:0048285	Organelle fission	45	34.1	3.50E-31	9.10E-02	PRC1, NEK2, KNTC1, TTK, CHEK1, AURKA, CEP55, PTTG1, AURKB, MTFR2, KIF2C, CDCA8, NCAPG, BUB1, RANBP1, TOP2A, CDCA5, CCNA2, ASPM, TRIP13, CDK1, KIF11, MKI67, DLGAP5, KIF15, TPX2, CENPF, NUSAP1, CENPE, CDC20, BIRC5, RACGAP1, UBE2C, TACC3, CDC25C, SMC4, NCAPD2, CCNB1, CCNB2, MAD2L1, ZWINT, CKS2, RUVBL1, CIT, UBE2S *(45)*	
**Analysis of Down-regulated DGEs**							
GO:0003008	System process	64	22.4	4.40E-08	2.30E-05	THRA, PTGS2, PTGS1, SOBP, FGF10, VCL, FOS, ACTG2, ATP2B4, DES, PRRT2, SLMAP, SERPINA3, LMOD1, ERRFI1, KCNMA1, KCND3, SGK1, PTGER3, ROCK2, CRYAB, SERPING1, NRXN1, SSPN, FLNA, EYA4, CHRM3, HSPB6, CD34, PDE5A, CELF2, AKAP6, JAM3, CASQ2, SGCA, CALD1, CAMK2G, CACNB2, TPM2, TPM1, KCNMB1, MYL9, ANXA6, SORBS1, PDE1C, SORBS2, DMD, CNN1, MYOM1, RCSD1, DTNA, IL6, ACTC1, ATP1A2, ITPR1, ABCC9, PLN, ITGA8, MYH11, HBEGF, GAMT, SYNM, MYLK, TMOD1 *(64)*	
GO:0023051	Regulation of signaling	64	22.4	1.20E-02	1.50E-01	RHOJ, RBPMS2, THRA, PTGS2, FGF9, LMO3, SNCA, PREX2, FGF10, RORA, KANK1, ATP2B4, SRPX, ARHGAP20, GPC3, PTGIS, ILK, SH3BGR, RHOB, PELI2, YAP1, FGF2, ERRFI1, EGR1, PID1, WLS, NRXN1, FLNA, SLIT3, NCAM1, HHEX, EYA4, RBPMS, PDE5A, PLA2G2A, AKAP6, GADD45B, CASQ2, SH3GL2, C3, ARHGEF25, IRAK3, PLCL1, SORBS1, DMD, PKD2, DIXDC1, IL6, NTF3, SYT11, ATP1A2, ITPR1, FZD7, NTRK3, LAMA2, ABCC9, EPHA7, ITGA8, PLN, HBEGF, TGFBR3, PTCH1, IGFBP2, ARHGAP10 *(64)*	
GO:0010646	Regulation of cell communication	63	22	1.30E-02	1.60E-01	RHOJ, RBPMS2, THRA, PTGS2, FGF9, LMO3, SNCA, PREX2, FGF10, RORA, KANK1, ATP2B4, SRPX, ARHGAP20, GPC3, PTGIS, ILK, SH3BGR, RHOB, PELI2, YAP1, FGF2, ERRFI1, EGR1, PID1, WLS, NRXN1, FLNA, SLIT3, NCAM1, HHEX, EYA4, RBPMS, PDE5A, PLA2G2A, AKAP6, GADD45B, CASQ2, SH3GL2, C3, ARHGEF25, IRAK3, PLCL1, SORBS1, DMD, PKD2, DIXDC1, IL6, NTF3, SYT11, ATP1A2, ITPR1, FZD7, NTRK3, LAMA2, EPHA7, ITGA8, PLN, HBEGF, TGFBR3, PTCH1, IGFBP2, ARHGAP10 *(63)*	
GO:0065009	Regulation of molecular function	62	21.7	6.20E-03	9.90E-02	FGF9, LEPR, SNCA, PREX2, FGF10, DDR2, FOS, ATP2B4, GPC3, ARHGAP20, PTGIS, ANG, SLMAP, ILK, SERPINA3, ITIH5, LRRFIP1, PI16, FGF2, ERRFI1, SGK1, ROCK2, CRYAB, SERPING1, NRXN1, FLNA, NCAM1, HHEX, PDE5A, AKAP6, GADD45B, CASQ2, C3, ARHGEF25, FHL1, CAMK2G, CACNB2, TPM2, TPM1, ALDH1A1, IRAK3, PLCL1, DMD, PKD2, TXNIP, IL6, ADARB1, NTF3, ACACB, ATP1A2, EPHA3, C2ORF40, NTRK3, FNBP1, EPHA7, PLN, TGFBR3, GFRA1, HBEGF, PTCH1, NFIB, ARHGAP10 *(62)*	
GO:0007399	Nervous system development	61	21.3	6.30E-06	9.60E-04	FGF9, PREX2, FGF10, RORA, KANK1, VCL, CKB, FOS, ATP2B4, PCP4, MAPT, ILK, YAP1, UNC5C, ATOH8, FGF2, BOC, PITX1, NR2F1, SGK1, WLS, NRXN1, NDNF, TACC1, PRELP, SLIT3, NCAM1, HHEX, CHRM3, PRDM6, CNTN1, JAM3, SH3GL2, FMOD, CAMK2G, ZBTB16, MEIS1, ITM2A, DMD, PKD2, LRFN5, DIXDC1, IL6, NTF3, UST, MAP1B, MAOB, NTNG1, AK4, FZD7, EPHA3, NTRK3, LAMA2, CSGALNACT1, EPHA7, ITGA8, GFRA1, PTCH1, ID4, NFIB, CDH11 *(61)*	
GO:0070887	Cellular response to chemical stimulus	60	21	2.70E-03	5.80E-02	RBPMS2, THRA, PTGS2, LMO3, FGF9, LEPR, SNCA, FERMT2, PTGS1, FGF10, RORA, KANK1, FOS, ATP2B4, GPC3, PTGIS, ILK, RHOB, YAP1, GLP2R, FGF2, ERRFI1, NR2F1, PID1, EGR1, ROCK2, FOSB, NDNF, SLIT3, NCAM1, HHEX, CHRM3, CD34, CLIC4, AKAP6, JAM3, CASQ2, FMOD, CAMK2G, NR3C2, TPM1, KCNMB1, IRAK3, SORBS1, PKD2, IL6, ATP1A2, PTGFR, FZD7, SOD3, EPHA3, NTRK3, PLSCR4, ITGA8, FBLN5, HIF3A, TGFBR3, HBEGF, PTCH1, IGFBP2 *(60)*	
GO:0010033	Response to organic substance	60	21	1.10E-02	1.40E-01	RBPMS2, THRA, PTGS2, LMO3, FGF9, LEPR, SNCA, FERMT2, FGF10, RORA, KANK1, FOS, ATP2B4, GPC3, PTGIS, ANG, ILK, GPX3, YAP1, GLP2R, FGF2, ERRFI1, NR2F1, PID1, KCNMA1, EGR1, ROCK2, CRYAB, FOSB, SLIT3, NCAM1, HHEX, CHRM3, AKAP6, CASQ2, FMOD, RBP1, CAMK2G, NR3C2, KCNMB1, IRAK3, SORBS1, TXNIP, IL6, ACTC1, MAOB, AFF3, ACACB, ATP1A2, PTGFR, FZD7, EPHA3, NTRK3, PLSCR4, PLN, ITGA8, TGFBR3, ABCC4, PTCH1, IGFBP2 *(60)*	
GO:0006928	Movement of cell or subcellular component	59	20.6	5.70E-08	2.70E-05	PTGS2, FGF10, DDR2, KANK1, VCL, KIF13A, ATP2B4, DES, ANG, MAPT, ILK, RHOB, UNC5C, ATOH8, FGF2, BOC, KCND3, SGK1, ROCK2, ACTN1, NRXN1, NEXN, PALLD, FLNA, NDNF, SLIT3, NCAM1, TNS1, CD34, CLIC4, JAM3, DST, SH3GL2, FMOD, CALD1, CACNB2, TPM2, TPM1, SORBS2, DMD, FYCO1, DIXDC1, IL6, ACTC1, ADARB1, NTF3, MAP1B, ATP1A2, EPHA3, NTRK3, LSP1, LAMA2, EPHA7, PLN, TGFBR3, HBEGF, MYLK, NFIB, TMOD1 *(59)*	
GO:0048468	Cell development	58	20.3	2.80E-06	6.60E-04	FERMT2, PREX2, KANK1, VCL, ANG, MAPT, ILK, YAP1, UNC5C, LMOD1, BOC, FGF2, NR2F1, SGK1, ROCK2, ACTN1, NRXN1, NEXN, FLNC, PALLD, FLNA, NDNF, SLIT3, PRELP, NCAM1, CLIC4, PDE5A, PYGO1, AKAP6, CNTN1, JAM3, CASQ2, FMOD, ZBTB16, TPM1, MEIS1, SORBS2, DMD, LRFN5, DIXDC1, IL6, ACTC1, NTF3, UST, MAP1B, NTNG1, FZD7, EPHA3, NTRK3, LAMA2, EPHA7, ITGA8, MYH11, TGFBR3, ID4, NFIB, CDH11, TMOD1 *(58)*	
GO:0009966	Regulation of signal transduction	58	20.3	1.20E-02	1.50E-01	RHOJ, RBPMS2, THRA, PTGS2, LMO3, FGF9, SNCA, PREX2, FGF10, RORA, KANK1, ATP2B4, SRPX, ARHGAP20, GPC3, PTGIS, ILK, SH3BGR, RHOB, PELI2, YAP1, ERRFI1, FGF2, PID1, EGR1, WLS, NRXN1, FLNA, SLIT3, EYA4, HHEX, RBPMS, PDE5A, PLA2G2A, AKAP6, GADD45B, CASQ2, SH3GL2, C3, ARHGEF25, IRAK3, SORBS1, DMD, PKD2, DIXDC1, IL6, NTF3, ITPR1, FZD7, NTRK3, EPHA7, ITGA8, PLN, TGFBR3, HBEGF, PTCH1, IGFBP2, ARHGAP10 *(58)*	
