# Supplementary material for: Identification of Key Biomarkers in Bladder Cancer: Evidence from a Bioinformatics Analysis
Source: Diagnostics (Basel). 2020 Jan 24;10(2):66. doi: 10.3390/diagnostics10020066 (PMC7168923; doi:10.3390/diagnostics10020066)
Supplement: Supplementary file 1 [file diagnostics-10-00066-s001.zip › Table S2.pdf]

Table S2. GOTERM_CC analysis. Analysis of total DEGs and the subgroups of up- and down-regulated DEGs. Numbers in brackets indicate count of DEGs.

ID	Description	Count in Gene set	Percent -age%	P-value	Benjamini	List of Genes	
						**Up-regulated**	**Down-regulated**
**Analysis of Total DGEs**							
GO:0043292	Contractile fiber	34	8.1	1.60E-16	6.50E-14	TPM3 SCO2 (2)	ARHGEF25 CALD1 FERMT2 PDLIM3 TPM2 TPM1 MYL9 VCL ATP2B4 DES SORBS2 DMD ILK LMOD1 MYOM1 ACTC1 CRYAB ACTN1 SYNPO2 FLNC PALLD NEXN FLNA ABCC9 SYNE1 PGM5 SVIL MYH11 SYNM DST CASQ2 TMOD (32)
GO:0030016	Myofibril	33	7.9	2.30E-16	6.50E-14	TPM3 SCO2 (2)	ARHGEF25 CALD1 FERMT2 PDLIM3 TPM2 TPM1 MYL9 VCL DES ATP2B4 SORBS2 DMD ILK LMOD1 MYOM1 ACTC1 CRYAB ACTN1 SYNPO2 FLNC PALLD NEXN FLNA ABCC9 SYNE1 PGM5 SVIL SYNMDST CASQ2 TMOD1 (31)
GO:0044449	Contractile fiber part	32	7.7	7.70E-16	1.50E-13	TPM3 (1)	ARHGEF25 FERMT2 PDLIM3 TPM2 TPM1 MYL9 VCL DES ATP2B4 SORBS2 DMD ILK LMOD1 MYOM1 ACTC1 CRYAB ACTN1 SYNPO2 FLNC PALLD NEXN FLNA ABCC9 SYNE1 PGM5 SVIL MYH11 SYNM DST CASQ2 TMOD1 (31)
GO:0030017	Sarcomere	28	6.7	2.20E-13	3.10E-11	TPM3 (1)	ARHGEF25 FERMT2 PDLIM3 TPM2 TPM1 MYL9 DES ATP2B4 SORBS2 DMD ILK LMOD1 MYOM1 ACTC1 CRYAB ACTN1 SYNPO2 FLNC PALLD NEXN FLNA SYNE1 ABCC9 PGM5 DST CASQ2 TMOD1 (44)
GO:0005819	Spindle	29	6.9	3.10E-09	3.60E-07	PRC1 NEK2 KNTC1 TTK AURKA AURKB CDCA8 ASPM CKAP2 CDK1 KIF11 DLGAP5 KIF15 TPX2 NUSAP1 CENPF BIRC5 CDC20 CENPE RACGAP1 TACC3 ECT2 CCNB1 MAD2L1 CKAP2L KIF20A (26)	PKD2 KLHL42 MAP7D3 (3)
GO:0030496	Midbody	19	4.5	6.20E-09	6.10E-07	CDK1 PRC1 NEK2 CENPF CENPE AURKA BIRC5 AURKB CEP55 RACGAP1 ECT2 CDCA8 ASPM KIF20A (14)	TACC1 KIF13A CLIC4 SVIL PTCH1 (5)
GO:0015630	Microtubule cytoskeleton	61	14.6	9.00E-09	7.50E-07	STIL PRC1 KNTC1 TTK AURKA AURKB KIF2C CDCA8 RANBP1 TOP2A TUBA1C ASPM CDK1 KIF11 KIF15 TPX2 NUSAP1 MCM2 TACC ECT2 MAD2L1 CKAP2L STMN1 RUVBL1 KIF26B NEK2 CHEK1 CEP55 TUBB NCAPG CKAP2 SHMT2 DLGAP5 RGS19 CENPF CENPE BIRC5 CDC20 RACGAP1 CCNB1 PLK4 CCNB2 PCNA KIF20A (44)	KIF13A MAPT SLMAP TUBB6 TUBA1A ROCK2 CRYAB TACC1 CLIC4 DST MAP7D3 SORBS1 PKD2 KLHL42 MAP1A MAP1B SVIL (17)
GO:0099513	Polymeric cytoskeletal fiber	45	10.8	2.40E-08	1.70E-06	PRC1 LMNB1 NEK2 KNTC1 AURKA AURKB TPM3 KIF2C TUBB TUBA1C ASPM CKAP2 CDK1 KIF11 KIF15 TPX2 RGS19 NUSAP1 CENPE BIRC5 RACGAP1 STMN1 KIF26B KIF20A (24)	FERMT2 TPM2 TPM1 KIF13A DES MAPT PKD2 TUBB6 LMOD1 TUBA1A RCSD1 ACTC1 MAP1A MAP1B ACTN1 PALLD FLNA SVIL SYNM DST TMOD1 (21)
GO:0099512	Supramolecular fiber	45	10.8	2.40E-08	1.70E-06	PRC1 LMNB1 NEK2 KNTC1 AURKA AURKB TPM3 KIF2C TUBB TUBA1C ASPM CKAP2 CDK1 KIF11 KIF15 TPX2 RGS19 NUSAP1 CENPE BIRC5 RACGAP1 STMN1 KIF26B KIF20A (24)	FERMT2 TPM2 TPM1 KIF13A DES MAPT PKD2 TUBB6 LMOD1 TUBA1A RCSD1 ACTC1 MAP1A MAP1B ACTN1 PALLD FLNA SVIL SYNM DST TMOD1 (21)
GO:0031674	I band	18	4.3	3.10E-08	1.80E-06		ACTC1 CRYAB FERMT2 PDLIM3 ACTN1 SYNPO2 PALLD NEXN FLNC FLNA MYL9 PGM5 DES ATP2B4 SORBS2 DMD DST CASQ2 (18)
**Analysis of Up-regulated DGEs**							
GO:0005654	Nucleoplasm	54	40.9	1.30E-08	3.40E-07	E2F2, PRC1, EZH2, AURKA, CBX2, AURKB, MCM10, CDCA8, HIST1H2BK, FANCI, TOP2A, CDCA5, CCNA2, CDK1, TPX2, MCM2, UBE2C, MCM5, NCAPD2, RRM2, SNRPB, RUVBL1, KPNA2, UBE2T, CKS1B, HMGB2, BLM, LMNB1, FOXM1, KIAA0101, CHEK1, TYMS, HJURP, BUB1, POLQ, SCO2, FEN1, ETV4, SIM2, TFDP1, RAD51AP1, HIST1H2BD, CENPF, BIRC5, CDC20, RACGAP1, CDC25C, SMC4, CCNB1, CCNB2, ILF2, PARPBP, PCNA, KIF20A *(54)*	
GO:0005829	Cytosol	50	37.9	3.30E-05	5.20E-04	STIL, PRC1, AP2S1, DTYMK, KNTC1, AURKA, PTTG1, AURKB, KIF2C, CDCA8, CDCA5, DHCR24, CDK1, KIF11, KIF15, TPX2, UBE2C, ECT2, NCAPD2, MAD2L1, ZWINT, RRM2, SNRPB, STMN1, KPNA2, ARL6IP1, NEK2, SNX8, DIAPH3, CHEK1, TK1, HMMR, TPM3, TYMS, NCAPG, PAFAH1B3, FASN, BUB1, CENPF, BIRC5, CDC20, CENPE, RACGAP1, CDC25C, SMC4, CCNB1, PLK4, CCNB2, BAX, CIT *(50)*	
GO:0015630	Microtubule cytoskeleton	44	33.3	1.90E-18	7.40E-16	STIL, PRC1, NEK2, KNTC1, TTK, CHEK1, AURKA, CEP55, AURKB, KIF2C, CDCA8, TUBB, NCAPG, RANBP1, TOP2A, TUBA1C, ASPM, CKAP2, CDK1, SHMT2, KIF11, DLGAP5, KIF15, TPX2, RGS19, CENPF, NUSAP1, CENPE, BIRC5, CDC20, MCM2, RACGAP1, TACC3, ECT2, CCNB1, PLK4, CCNB2, MAD2L1, CKAP2L, PCNA, STMN1, RUVBL1, KIF26B, KIF20A *(44)*	
GO:0005694	Chromosome	41	31.1	2.00E-18	3.90E-16	HMGB2, BLM, NEK2, EZH2, KNTC1, TTK, CBX2, CHEK1, AURKA, AURKB, MCM10, KIF2C, CDCA8, HIST1H2BK, HJURP, NCAPG, BUB1, HIST3H2A, POLQ, TOP2A, CDCA5, FEN1, CDK1, RAD51AP1, HIST1H2BD, MKI67, CENPF, NUSAP1, CENPE, BIRC5, MCM2, MCM5, SMC4, NCAPD2, CCNB1, PLK4, MAD2L1, ZWINT, PARPBP, PCNA, RUVBL1 *(41)*	
GO:0044427	Chromosomal part	37	28	8.70E-17	8.70E-15	HMGB2, BLM, NEK2, EZH2, KNTC1, TTK, CBX2, CHEK1, AURKA, AURKB, MCM10, KIF2C, CDCA8, HIST1H2BK, HJURP, NCAPG, BUB1, HIST3H2A, CDCA5, FEN1, CDK1, RAD51AP1, 37HIST1H2BD, MKI67, CENPF, CENPE, BIRC5, MCM2, MCM5, SMC4, NCAPD2, CCNB1, MAD2L1, ZWINT, PARPBP, PCNA, RUVBL1 *(37)*	
GO:0005819	Spindle	26	19.7	4.40E-18	5.70E-16	PRC1, NEK2, KNTC1, TTK, AURKA, AURKB, CDCA8, ASPM, CKAP2, CDK1, KIF11, DLGAP5, KIF15, TPX2, NUSAP1, CENPF, BIRC5, CDC20, CENPE, RACGAP1, TACC3, ECT2, CCNB1, MAD2L1, CKAP2L, KIF20A *(26)*	

GO:0098687	Chromosomal region	25	18.9	1.20E-15	7.90E-14	CDK1, MKI67, BLM, NEK2, KNTC1, CENPF, TTK, CENPE, BIRC5, CHEK1, AURKA, MCM2, AURKB, MCM5, NCAPD2, CCNB1, KIF2C, CDCA8, MAD2L1, HJURP, ZWINT, PCNA, BUB1, CDCA5, FEN1
GO:0000228	Nuclear chromosome	24	18.2	2.20E-10	7.20E-09	CDK1, HMGB2, RAD51AP1, HIST1H2BD, BLM, NEK2, EZH2, BIRC5, CHEK1, AURKA, MCM2, AURKB, MCM10, MCM5, NCAPD2, CCNB1, HIST1H2BK, BUB1, PCNA, HIST3H2A, RUVBL1, CDCA5, TOP2A, FEN1
GO:0099512	Supramolecular fiber	24	18.2	2.10E-08	4.80E-07	CKAP2, CDK1, KIF11, LMNB1, PRC1, NEK2, KIF15, TPX2, KNTC1, NUSAP1, RGS19, BIRC5, AURKA, CENPE, AURKB, RACGAP1, TPM3, KIF2C, TUBB, STMN1, ASPM, TUBA1C, KIF26B, KIF20A
GO:0099513	Polymeric cytoskeletal fiber	24	18.2	2.10E-08	4.80E-07	CKAP2, CDK1, KIF11, LMNB1, PRC1, NEK2, KIF15, TPX2, KNTC1, NUSAP1, RGS19, BIRC5, AURKA, CENPE, AURKB, RACGAP1, TPM3, KIF2C, TUBB, STMN1, ASPM, TUBA1C, KIF26B, KIF20A
GO:0005654	Nucleoplasm	54	40.9	1.30E-08	3.40E-07	E2F2, PRC1, EZH2, AURKA, CBX2, AURKB, MCM10, CDCA8, HIST1H2BK, FANCI, TOP2A, CDCA5, CCNA2, CDK1, TPX2, MCM2, UBE2C, MCM5, NCAPD2, RRM2, SNRPB, RUVBL1, KPNA2, UBE2T, CKS1B, HMGB2, BLM, LMNB1, FOXM1, KIAA0101, CHEK1, TYMS, HJURP, BUB1, POLQ, SCO2, FEN1, ETV4, SIM2, TFDP1, RAD51AP1, HIST1H2BD, CENPF, BIRC5, CDC20, RACGAP1, CDC25C, SMC4, CCNB1, CCNB2, ILF2, PARPBP, PCNA, KIF20A
GO:0005829	Cytosol	50	37.9	3.30E-05	5.20E-04	STIL, PRC1, AP2S1, DTYMK, KNTC1, AURKA, PTTG1, AURKB, KIF2C, CDCA8, CDCA5, DHCR24, CDK1, KIF11, KIF15, TPX2, UBE2C, ECT2, NCAPD2, MAD2L1, ZWINT, RRM2, SNRPB, STMN1, KPNA2, ARL6IP1, NEK2, SNX8, DIAPH3, CHEK1, TK1, HMMR, TPM3, TYMS, NCAPG, PAFAH1B3, FASN, BUB1, CENPF, BIRC5, CDC20, CENPE, RACGAP1, CDC25C, SMC4, CCNB1, PLK4, CCNB2, BAX, CIT
**Analysis of Down-regulated DGEs**						
GO:0005576	Extracellular region	104	36.4	2.60E-04	4.10E-03	SNCA, PTGS1, FGF10, VCL, ACTG2, PTGIS, RAB23, CFD, PI16, SPON1, BST1, SPARCL1, CRYAB, CRTAP, ACTN1, MGP, SERPING1, PRELP, NCAM1, CD34, PLA2G2A, MFAP4, DST, SH3GL2, MAMDC2, ADAMTSL3, PAMR1, P3H2, OSBPL1A, FGL2, SBSPON, VSTM4, BHMT2, NTF3, MAOB, AK4, CSRP1, IGIP, EPHA3, LAMA2, C2ORF40, HSPA12A, MYH11, TGFBR3, ANTXR2, MYLK, KCTD12, RHOJ, FGF9, LEPR, DDR2, DSTN, CKB, DES, GPC3, ANG, GPX3, SERPINA3, RHOB, TUBB6, ITIH5, TUBA1A, FGF2, KCNMA1, STX2, CPXM2, PKDCC, WLS, NDNF, FLNA, SLIT3, CLIC4, CNTN1, JAM3, C7, FMOD, C3, C1S, ITM2A, ANXA6, ALDH1A1, IGSF10, GLIPR1, PKD2, ADAMTS10, DPT, ACTC1, IL6, SVEP1, CREB5, ATP1A2, PTGFR, SOD3, PLAC9, LSP1, PLSCR4, FBLN5, AOX1, HBEGF, GFRA1, GAMT, IGFBP2, PYGB, CDH11
GO:0044421	Extracellular region part	90	31.5	3.40E-04	5.20E-03	RHOJ, FGF9, SNCA, PTGS1, FGF10, DDR2, DSTN, CKB, VCL, ACTG2, DES, GPC3, PTGIS, ANG, GPX3, SERPINA3, RAB23, RHOB, TUBB6, TUBA1A, CFD, PI16, FGF2, SPON1, KCNMA1, STX2, CRYAB, SPARCL1, BST1, CRTAP, ACTN1, MGP, CPXM2, SERPING1, WLS, FLNA, NDNF, PRELP, SLIT3, NCAM1, CLIC4, CD34, PLA2G2A, CNTN1, MFAP4, JAM3, DST, SH3GL2, C7, FMOD, MAMDC2, C3, ADAMTSL3, C1S, ITM2A, P3H2, ALDH1A1, ANXA6, OSBPL1A, PKD2, ADAMTS10, FGL2, DPT, SBSPON, BHMT2, ACTC1, IL6, MAOB, CREB5, CSRP1, ATP1A2, AK4, SOD3, C2ORF40, LSP1, LAMA2, HSPA12A, PLSCR4, FBLN5, AOX1, MYH11, HBEGF, GFRA1, TGFBR3, GAMT, IGFBP2, MYLK, CDH11, PYGB, KCTD12
GO:0031988	Membrane-bound vesicle	84	29.4	5.80E-04	7.50E-03	RHOJ, FGF9, SNCA, PTGS1, DDR2, DSTN, VCL, CKB, ACTG2, DES, GPC3, CD93, ANG, GPX3, SERPINA3, RAB23, RHOB, TUBB6, TUBA1A, CFD, PI16, KCNMA1, STX2, CRYAB, SPARCL1, BST1, ACTN1, MGP, SERPING1, PCDH7, WLS, NRXN1, SSPN, FLNA, PRELP, NCAM1, CLIC4, PLA2G2A, CNTN1, MFAP4, DST, SH3GL2, C7, C3, CAMK2G, C1S, ITM2A, ALDH1A1, ANXA6, OSBPL1A, PKD2, GALNT15, FGL2, DPT, BHMT2, ACTC1, NTF3, SYT11, MAOB, CREB5, CSRP1, AK4, SOD3, ITPR1, C2ORF40, LSP1, LAMA2, FNBP1, HSPA12A, PLSCR4, FBLN5, AOX1, MYH11, HBEGF, ABCC4, GFRA1, TGFBR3, PTCH1, GAMT, IGFBP2, MYLK, CDH11, PYGB, KCTD12
GO:1903561	Extracellular vesicle	70	24.5	3.90E-04	5.70E-03	RHOJ, FGF9, PTGS1, DDR2, DSTN, VCL, CKB, ACTG2, DES, GPC3, ANG, GPX3, SERPINA3, RAB23, TUBB6, RHOB, TUBA1A, CFD, PI16, KCNMA1, STX2, SPARCL1, BST1, CRYAB, ACTN1, MGP, SERPING1, WLS, FLNA, PRELP, NCAM1, CLIC4, PLA2G2A, CNTN1, MFAP4, DST, SH3GL2, C7, C3, C1S, ITM2A, ALDH1A1, ANXA6, OSBPL1A, PKD2, FGL2, DPT, ACTC1, BHMT2, MAOB, CREB5, CSRP1, AK4, ATP1A2, SOD3, LSP1, LAMA2, HSPA12A, PLSCR4, FBLN5, AOX1, MYH11, TGFBR3, GFRA1, GAMT, IGFBP2, MYLK, PYGB, KCTD12, CDH11
GO:0043230	Extracellular organelle	70	24.5	3.90E-04	5.60E-03	RHOJ, FGF9, PTGS1, DDR2, DSTN, VCL, CKB, ACTG2, DES, GPC3, ANG, GPX3, SERPINA3, RAB23, TUBB6, RHOB, TUBA1A, CFD, PI16, KCNMA1, STX2, SPARCL1, BST1, CRYAB, ACTN1, MGP, SERPING1, WLS, FLNA, PRELP, NCAM1, CLIC4, PLA2G2A, CNTN1, MFAP4, DST, SH3GL2, C7, C3, C1S, ITM2A, ALDH1A1, ANXA6, OSBPL1A, PKD2, FGL2, DPT, ACTC1, BHMT2, MAOB, CREB5, CSRP1, AK4, ATP1A2, SOD3, LSP1, LAMA2, HSPA12A, PLSCR4, FBLN5, AOX1, MYH11, TGFBR3, GFRA1, GAMT, IGFBP2, MYLK, PYGB, KCTD12, CDH11
GO:0070062	Extracellular exosome	69	24.1	5.70E-04	7.90E-03	RHOJ, FGF9, PTGS1, DDR2, DSTN, VCL, CKB, ACTG2, DES, GPC3, ANG, GPX3, SERPINA3, RAB23, TUBB6, RHOB, TUBA1A, CFD, PI16, KCNMA1, STX2, SPARCL1, BST1, CRYAB, MGP, ACTN1, SERPING1, WLS, FLNA, PRELP, NCAM1, CLIC4, PLA2G2A, CNTN1, MFAP4, DST, SH3GL2, C7, C3, C1S, ITM2A, ALDH1A1, ANXA6, OSBPL1A, PKD2, FGL2, DPT, ACTC1, BHMT2, MAOB, CREB5, CSRP1, AK4, SOD3, LSP1, LAMA2, HSPA12A, PLSCR4, FBLN5, AOX1, MYH11, TGFBR3, GFRA1, GAMT, IGFBP2, MYLK, PYGB, KCTD12, CDH11
GO:0030054	Cell junction	48	16.8	1.50E-06	3.70E-05	FHL1, CALD1, SNCA, FERMT2, DDR2, VCL, ANXA6, DES, PRRT2, SORBS1, SORBS2, DMD, ILK, PKD2, RHOB, CNN1, UNC5C, LRRFIP1, DTNA, DIXDC1, ACTC1, LPP, SYT11, MAP1B, ACTN1, SYNPO2, CSRP1, NRXN1, ATP1A2, NEXN, FLNC, PALLD, SSPN, FLNA, TMEM47, TNS1, PGM5, CHRM3, STXBP6, CLIC4, SVIL, ITGA8, AKAP6, SYNM, DST, JAM3, SGCA, KCTD12
GO:0005615	Extracellular space	36	12.6	1.50E-02	1.10E-01	FMOD, C3, FGF9, SNCA, FGF10, C1S, CKB, ACTG2, PTGIS, GPC3, ANG, GPX3, SERPINA3, FGL2, CFD, FGF2, DPT, SPON1, IL6, ACTC1, STX2, SPARCL1, CRTAP, CPXM2, ACTN1, SERPING1, SOD3, SLIT3, PRELP, C2ORF40, FBLN5, PLA2G2A, HBEGF, TGFBR3, IGFBP2, JAM3 243
GO:0097458	Neuron part	35	12.2	8.10E-03	6.80E-02	PTGS2, CAMK2G, SNCA, PTGS1, FOS, ATP2B4, FAM107A, ANG, DMD, MAPT, ILK, UNC5C, BOC, DTNA, KCND3, STX2, CRYAB, SYT11, RBFOX3, MAP1B, WLS, ATP1A2, NRXN1, PALLD, FLNA, ITPR1, LAMA2, EPHA7, CHRM3, ITGA8, SYNM, PTCH1, DST, KCTD12, NFIB
GO:0043292	Contractile fiber	32	11.2	5.90E-20	1.20E-17	ARHGEF25, CALD1, FERMT2, PDLIM3, TPM2, TPM1, MYL9, VCL, ATP2B4, DES, SORBS2, DMD, ILK, LMOD1, MYOM1, ACTC1, CRYAB, ACTN1, SYNPO2, FLNC, PALLD, NEXN, FLNA, ABCC9, SYNE1, PGM5, SVIL, MYH11, SYNM, DST, CASQ2, TMOD1
