# Supplementary material for: Identification of Key Biomarkers in Bladder Cancer: Evidence from a Bioinformatics Analysis
Source: Diagnostics (Basel). 2020 Jan 24;10(2):66. doi: 10.3390/diagnostics10020066 (PMC7168923; doi:10.3390/diagnostics10020066)
Supplement: Supplementary file 1 [file diagnostics-10-00066-s001.zip › Table S3.pdf]

Table S3. GOTERM_MF analysis. Analysis of total DEGs and the subgroups of up- and down-regulated DEGs. Numbers in brackets indicate count of DEGs.

ID	Description	Count in Gene set	Percent -age%	P-value	Benjamini	List of Genes	
						Up-regulated	Down-regulated
Analysis of Total DGEs							
GO:0008092	Cytoskeletal protein binding	58	13.9	5.50E-13	5.00E-10	PRC1 KIF2C KIF11 KIF15 NUSAP1 STMN1 KIF26B DIAPH3 TPM3 CENPE BIRC5 RACGAP1 KIF20A (13)	SNCA PDLIM3 DSTN VCL KIF13A DES ANG MAPT LMOD1 KCNMA1 CRYAB ACTN1 NEXN FLNC PALLD FLNA TNS1 TAGLN DST MAP7D3 CALD1 CACNB2 TPM2 TPM1 MYL9 SORBS1 SORBS2 DMD PKD2 CNN1 RCSD1 DIXDC1 ACTC1 MAP1A SYT11 MAP1B SYNPO2 REEP1 LSP1 SYNE1 SVIL MYH11 SYNM MYLK TMOD1 (45)
GO:0003779	Actin binding	31	7.4	2.00E-08	9.20E-06	DIAPH3 TPM3 (2)	CALD1 CACNB2 TPM2 TPM1 DSTN VCL SORBS1 ANG DMD LMOD1 CNN1 RCSD1 KCNMA1 DIXDC1 ACTN1 SYNPO2 FLNC PALLD NEXN FLNA LSP1 SYNE1 TNS1 TAGLN SVIL MYH11 DST MYLK TMOD1 (29)
GO:0008307	Structural constituent of muscle	11	2.6	3.50E-08	1.10E-05	TPM3 (1)	SORBS2 DMD PDLIM3 MYH11 SYNM MYOM1 NEXN TPM2 TPM1 MYL9 (10)
GO:0097367	Carbohydrate derivative binding	88	21.1	1.10E-06	2.40E-04	DTYMK TTK AURKA AURKB PAK6 KIF2C TOP2A TUBA1C CDK1 KIF11 KIF15 TPX2 MCM2 UBE2C MCM5 LAMC2 RUVBL1 UBE2S UBE2T MELK KIF26B BLM NEK2 CHEK1 HMMR TK1 TUBB BUB1 POLQ TRIP13 MKI67 CENPE SMC4 PLK4 ILF2 CIT KIF20A (37)	P RHOJ FGF9 FGF10 DDR2 CKB VCL KIF13A ACTG2 ATP2B4 GPC3 ANG ILK RAB23 RHOB TUBB6 TUBA1A FGF2 SGK1 ROCK2 PKDCC FLNA NDNF PRELP SLIT3 PDE5A CNTN1 ABCA8 FMOD RERGL CAMK2G ABCA6 ANXA6 IRAK3 DMD ACTC1 ATP1A2 AK4 ACACB SOD3 EPHA3 NTRK3 ABCC9 EPHA7 HSPA12A RAB30 MYH11 HBEGF ABCC4 TGFBR3 PTCH1 MYLK 1 (51)
GO:0008017	Microtubule binding	19	4.5	4.60E-06	8.30E-04	KIF11 PRC1 KIF15 NUSAP1 BIRC5 CENPE RACGAP1 KIF2C KIF26B KIF20A (10)	CRYAB MAP1A MAP1B SNCA REEP1 KIF13A MAPT DST MAP7D3 (9)
GO:0015631	Tubulin binding	22	5.3	6.90E-06	1.00E-03	KIF11 PRC1 KIF15 NUSAP1 BIRC5 CENPE RACGAP1 KIF2C STMN1 KIF26B KIF20A (11)	DIXDC1 CRYAB SYT11 MAP1A SNCA MAP1B REEP1 KIF13A MAPT DST MAP7D3 (11)
GO:0051015	Actin filament binding	14	3.3	1.40E-05	1.60E-03	TPM3 (1)	ACTN1 CACNB2 TPM2 NEXN FLNC TPM1 FLNA DSTN SYNE1 SVIL MYH11 RCSD1 TMOD1 (13)
GO:0042802	Identical protein binding	58	13.9	1.30E-05	1.60E-03	PRC1 MMP9 TTK MCM10 FAP TOP2A ECT2 PYCR1 INHBA MAD2L1 CTSC ARL6IP1 SNX8 TK1 TYMS COL7A1 HJURP FASN PAFAH1B3 TRIP13 SHMT2 CENPF BIRC5 PLK4 BAX PCNA (26)	RBPMS2 PTGS2 SNCA DES ANG LRRFIP1 CRYAB ACTN1 FLNA HHEX RBPMS HSPB6 PRDM6 JAM3 DST CASQ2 SH3GL2 CAMK2G ZBTB16 C1S ANXA6 IRAK3 PKD2 MYOM1 SYT11 MAOB FNBP1 SYNE1 PLN FBLN5 ZBTB4 DPYD (32)
GO:0046983	Protein dimerization activity	50	12	4.80E-05	4.80E-03	E2F2 TTK TYMS HIST1H2BK FAP PAFAH1B3 FASN HIST3H2A TOP2A SIM2 HIST1H2BD CENPF BIRC5 ECT2 SMC4 INHBA MAD2L1 BAX (18)	RBPMS2 PTGS2 CAMK2G ZBTB16 MEIS1 ANXA6 FOS IRAK3 ANG PKD2 ATOH8 MYOM1 LRRFIP1 STX2 CRYAB SYT11 MAOB ACTN1 FLNA HHEX RBPMS SYNE1 HSPB6 PRDM6 FBLN5 ZBTB4 HIF3A ID4 DPYD DST JAM3 CASQ2 (32)
GO:0042803	Protein homodimerization activity	36	8.6	5.50E-05	5.00E-03	TTK TYMS FAP FASN TOP2A CENPF BIRC5 ECT2 MAD2L1 BAX (10)	RBPMS2 PTGS2 CAMK2G ZBTB16 ANXA6 IRAK3 ANG PKD2 MYOM1 LRRFIP1 CRYAB SYT11 MAOB ACTN1 FLNA HHEX RBPMS SYNE1 HSPB6 PRDM6 FBLN5 ZBTB4 DPYD DST JAM3 CASQ2 (26)
Analysis of Up-regulated DGEs							
GO:1901363	Heterocyclic compound binding	61	46.2	2.60E-03	4.60E-02	TM7SF2, E2F2, EZH2, DTYMK, TTK, AURKA, CBX2, AURKB, MCM10, PAK6, KIF2C, HIST1H2BK, FANCI, HIST3H2A, TOP2A, TUBA1C, DHCR24, CDK1, KIF11, OTX1, KIF15, TPX2, NUSAP1, MCM2, UBE2C, MCM5, SNRPB, RUVBL1, KPNA2, UBE2S, MELK, UBE2T, KIF26B, HMGB2, BLM, NEK2, FOXM1, CHEK1, TK1, TYMS, TUBB, HJURP, BUB1, FASN, POLQ, FEN1, ETV4, SIM2, TFDP1, TRIP13, RAD51AP1, HIST1H2BD, MKI67, CENPE, SMC4, PLK4, ILF2, PARPBP, PCNA, CIT, KIF20A *(61)*	
GO:0097159	Organic cyclic compound binding	61	46.2	3.80E-03	5.90E-02	TM7SF2, E2F2, EZH2, DTYMK, TTK, AURKA, CBX2, AURKB, MCM10, PAK6, KIF2C, HIST1H2BK, FANCI, HIST3H2A, TOP2A, TUBA1C, DHCR24, CDK1, KIF11, OTX1, KIF15, TPX2, NUSAP1, MCM2, UBE2C, MCM5, SNRPB, RUVBL1, KPNA2, UBE2S, MELK, UBE2T, KIF26B, HMGB2, BLM, NEK2, FOXM1, CHEK1, TK1, TYMS, TUBB, HJURP, BUB1, FASN, POLQ, FEN1, ETV4, SIM2, TFDP1, TRIP13, RAD51AP1, HIST1H2BD, MKI67, CENPE, SMC4, PLK4, ILF2, PARPBP, PCNA, CIT, KIF20A *(61)*	
GO:0000166	Nucleotide binding	39	29.5	2.50E-06	1.00E-04	TM7SF2, BLM, NEK2, DTYMK, TTK, CHEK1, AURKA, AURKB, TK1, PAK6, TYMS, KIF2C, TUBB, FASN, BUB1, POLQ, TOP2A, TUBA1C, DHCR24, TRIP13, CDK1, KIF11, MKI67, KIF15, TPX2, CENPE, MCM2, UBE2C, MCM5, SMC4, PLK4, ILF2, RUVBL1, CIT, UBE2S, MELK, UBE2T, KIF26B, KIF20A *(39)*	
GO:1901265	Nucleoside phosphate binding	39	29.5	2.50E-06	9.30E-05	TM7SF2, BLM, NEK2, DTYMK, TTK, CHEK1, AURKA, AURKB, TK1, PAK6, TYMS, KIF2C, TUBB, FASN, BUB1, POLQ, TOP2A, TUBA1C, DHCR24, TRIP13, CDK1, KIF11, MKI67, KIF15, TPX2, CENPE, MCM2, UBE2C, MCM5, SMC4, PLK4, ILF2, RUVBL1, CIT, UBE2S, MELK, UBE2T, KIF26B, KIF20A *(39)*	
GO:0036094	Small molecule binding	39	29.5	1.40E-05	4.10E-04	TM7SF2, BLM, NEK2, DTYMK, TTK, CHEK1, AURKA, AURKB, TK1, PAK6, TYMS, KIF2C, TUBB, FASN, BUB1, POLQ, TOP2A, TUBA1C, DHCR24, TRIP13, CDK1, KIF11, MKI67, KIF15, TPX2, CENPE, MCM2, UBE2C, MCM5, SMC4, PLK4, ILF2, RUVBL1, CIT, UBE2S, MELK, UBE2T, KIF26B, KIF20A *(39)*	
GO:0019899	Enzyme binding	37	28	1.50E-08	7.30E-06	CKS1B, PRC1, LMNB1, NEK2, FOXM1, DIAPH3, AURKA, MCM10, TUBB, FANCI, FAP, MARVELD3, RANBP1, TOP2A, CCNA2, DHCR24, KIF11, TPX2, RGS19, CST1, CDC20, BIRC5, MCM2, RACGAP1, CDC25C, UBE2C, ECT2, CCNB1, CST6, PCNA, CKS2, CTSC, CIT, UBE2S, KPNA2, UBE2T, KIF20A *(37)*	

**GO:0097367**	**Carbohydrate derivative binding**	**37**	**28**	**4.10E-06**	**1.40E-04**	**BLM, NEK2, DTYMK, TTK, CHEK1, AURKA, AURKB, HMMR, TK1, PAK6, KIF2C, TUBB, BUB1, POLQ, TOP2A, TUBA1C, TRIP13, CDK1, KIF11, MKI67, KIF15, TPX2, CENPE, MCM2, UBE2C, MCM5, SMC4, PLK4, ILF2, LAMC2, RUVBL1, CIT, UBE2S, MELK, UBE2T, KIF26B, KIF20A**
**GO:0035639**	**Purine ribonucleoside triphosphate binding**	**35**	**26.5**	**3.50E-07**	**3.40E-05**	**BLM, NEK2, DTYMK, TTK, CHEK1, AURKA, AURKB, TK1, PAK6, KIF2C, TUBB, BUB1, POLQ, TOP2A, TUBA1C, TRIP13, CDK1, KIF11, MKI67, KIF15, TPX2, CENPE, MCM2, UBE2C, MCM5, SMC4, PLK4, ILF2, RUVBL1, CIT, UBE2S, MELK, UBE2T, KIF26B, KIF20A**
**GO:0032550**	**Purine ribonucleoside binding**	**35**	**26.5**	**3.90E-07**	**3.20E-05**	**BLM, NEK2, DTYMK, TTK, CHEK1, AURKA, AURKB, TK1, PAK6, KIF2C, TUBB, BUB1, POLQ, TOP2A, TUBA1C, TRIP13, CDK1, KIF11, MKI67, KIF15, TPX2, CENPE, MCM2, UBE2C, MCM5, SMC4, PLK4, ILF2, RUVBL1, CIT, UBE2S, MELK, UBE2T, KIF26B, KIF20A**
**GO:0001883**	**Purine nucleoside binding**	**35**	**26.5**	**4.10E-07**	**2.80E-05**	**BLM, NEK2, DTYMK, TTK, CHEK1, AURKA, AURKB, TK1, PAK6, KIF2C, TUBB, BUB1, POLQ, TOP2A, TUBA1C, TRIP13, CDK1, KIF11, MKI67, KIF15, TPX2, CENPE, MCM2, UBE2C, MCM5, SMC4, PLK4, ILF2, RUVBL1, CIT, UBE2S, MELK, UBE2T, KIF26B, KIF20A**
**Analysis of Down-regulated DGEs**						
**GO:0043167**	**Ion binding**	**92**	**32.2**	**2.50E-03**	**1.10E-01**	**THRA, PTGS2, LMO3, SNCA, PTGS1, PDLIM3, SOBP, ADH1B, RORA, ATP2B4, PTGIS, CD93, ANG, NR2F1, SPON1, EGR1, KCNMA1, KCND3, CRYAB, ROCK2, SPARCL1, RUNX1T1, ACTN1, MGP, CPXM2, PCDH7, NRXN1, SLIT3, EYA4, PGM5, CHRM3, ZNF135, CD34, PRDM6, PDE5A, PYGO1, PLA2G2A, ZFPM2, DST, CASQ2, SGCA, ADAMTSL3, FHL1, PAMR1, NR3C2, C1S, ZBTB16, MYL9, P3H2, ANXA6, IRAK3, PRUNE2, FAT3, PDE1C, SORBS2, DMD, PKD2, GALNT15, PLCD4, ADAMTS10, FYCO1, DTNA, BHMT2, ADARB1, SVEP1, LPP, SYT11, PDZRN4, ZNF25, CREB5, PDZRN3, CSRP1, ACACB, ATP1A2, SOD3, ITPR1, MSRB3, CSGALNACT1, ZFHX4, RNF150, PLSCR4, FBLN5, ITGA8, PRICKLE2, CDH19, AOX1, ZBTB4, ANTXR2, DPYD, MYLK, CDH11, PYGB**
**GO:0043169**	**Cation binding**	**90**	**31.5**	**1.60E-03**	**8.80E-02**	**THRA, PTGS2, LMO3, SNCA, PTGS1, PDLIM3, SOBP, ADH1B, RORA, ATP2B4, PTGIS, CD93, ANG, NR2F1, SPON1, EGR1, KCNMA1, KCND3, CRYAB, ROCK2, SPARCL1, RUNX1T1, ACTN1, MGP, CPXM2, PCDH7, NRXN1, SLIT3, EYA4, PGM5, CHRM3, ZNF135, PRDM6, PDE5A, PYGO1, PLA2G2A, ZFPM2, DST, CASQ2, SGCA, ADAMTSL3, FHL1, PAMR1, NR3C2, C1S, ZBTB16, MYL9, P3H2, ANXA6, IRAK3, PRUNE2, FAT3, PDE1C, SORBS2, DMD, PKD2, GALNT15, PLCD4, ADAMTS10, FYCO1, DTNA, BHMT2, ADARB1, SVEP1, LPP, SYT11, PDZRN4, ZNF25, CREB5, PDZRN3, CSRP1, ACACB, ATP1A2, SOD3, ITPR1, MSRB3, CSGALNACT1, ZFHX4, RNF150, PLSCR4, FBLN5, ITGA8, PRICKLE2, CDH19, AOX1, ZBTB4, ANTXR2, DPYD, MYLK, CDH11**
**GO:0046872**	**Metal ion binding**	**89**	**31.1**	**1.70E-03**	**8.80E-02**	**THRA, PTGS2, LMO3, SNCA, PTGS1, PDLIM3, SOBP, ADH1B, RORA, ATP2B4, PTGIS, CD93, ANG, NR2F1, SPON1, EGR1, KCNMA1, KCND3, CRYAB, ROCK2, SPARCL1, RUNX1T1, ACTN1, MGP, CPXM2, PCDH7, NRXN1, SLIT3, EYA4, PGM5, ZNF135, PRDM6, PDE5A, PYGO1, PLA2G2A, ZFPM2, DST, CASQ2, SGCA, ADAMTSL3, FHL1, PAMR1, NR3C2, C1S, ZBTB16, MYL9, P3H2, ANXA6, IRAK3, PRUNE2, FAT3, PDE1C, SORBS2, DMD, PKD2, GALNT15, PLCD4, ADAMTS10, FYCO1, DTNA, BHMT2, ADARB1, SVEP1, LPP, SYT11, PDZRN4, ZNF25, CREB5, PDZRN3, CSRP1, ACACB, ATP1A2, SOD3, ITPR1, MSRB3, CSGALNACT1, ZFHX4, RNF150, PLSCR4, FBLN5, ITGA8, PRICKLE2, CDH19, AOX1, ZBTB4, ANTXR2, DPYD, MYLK, CDH11**
**GO:0097367**	**Carbohydrate derivative binding**	**51**	**17.8**	**8.60E-03**	**2.40E-01**	**RHOJ, FGF9, FGF10, DDR2, VCL, CKB, KIF13A, ACTG2, ATP2B4, GPC3, ANG, ILK, RAB23, TUBB6, RHOB, TUBA1A, FGF2, SGK1, ROCK2, PKDCC, NDNF, FLNA, SLIT3, PRELP, PDE5A, CNTN1, ABCA8, FMOD, RERGL, CAMK2G, ABCA6, ANXA6, IRAK3, DMD, ACTC1, ACACB, AK4, ATP1A2, SOD3, EPHA3, NTRK3, ABCC9, HSPA12A, EPHA7, RAB30, MYH11, TGFBR3, ABCC4, HBEGF, PTCH1, MYLK**
**GO:0008092**	**Cytoskeletal protein binding**	**45**	**15.7**	**3.30E-12**	**2.30E-09**	**CALD1, SNCA, PDLIM3, CACNB2, TPM2, TPM1, DSTN, MYL9, VCL, KIF13A, DES, SORBS1, ANG, SORBS2, DMD, MAPT, PKD2, CNN1, LMOD1, RCSD1, KCNMA1, DIXDC1, ACTC1, CRYAB, SYT11, MAP1A, MAP1B, ACTN1, SYNPO2, REEP1, NEXN, PALLD, FLNC, FLNA, LSP1, SYNE1, TNS1, TAGLN, SVIL, MYH11, SYNM, DST, MAP7D3, MYLK, TMOD1**
**GO:0044877**	**Macromolecular complex binding**	**33**	**11.5**	**1.00E-02**	**2.50E-01**	**THRA, CACNB2, TPM2, TPM1, MEIS1, DDR2, DSTN, FOS, SORBS1, MAPT, ILK, YAP1, RCSD1, EGR1, SVEP1, SPARCL1, CRTAP, ACTN1, FLNC, NEXN, FLNA, HHEX, SYNE1, FBLN5, SVIL, PYGO1, MYH11, TGFBR3, PTCH1, SYNM, DST, JAM3, TMOD1**
**GO:0046983**	**Protein dimerization activity**	**32**	**11.2**	**3.40E-03**	**1.40E-01**	**RBPMS2, PTGS2, CAMK2G, ZBTB16, MEIS1, ANXA6, FOS, IRAK3, ANG, PKD2, ATOH8, MYOM1, LRRFIP1, STX2, CRYAB, SYT11, MAOB, ACTN1, FLNA, HHEX, RBPMS, SYNE1, HSPB6, PRDM6, FBLN5, HIF3A, ZBTB4, ID4, DPYD, DST, JAM3, CASQ2**
**GO:0042802**	**Identical protein binding**	**32**	**11.2**	**2.80E-02**	**4.60E-01**	**RBPMS2, PTGS2, CAMK2G, SNCA, ZBTB16, C1S, ANXA6, IRAK3, DES, ANG, PKD2, MYOM1, LRRFIP1, CRYAB, SYT11, MAOB, ACTN1, FLNA, HHEX, RBPMS, SYNE1, FNBP1, HSPB6, PRDM6, PLN, FBLN5, ZBTB4, DPYD, DST, JAM3, CASQ2, SH3GL2**
**GO:0003779**	**Actin binding**	**29**	**10.1**	**4.70E-11**	**1.60E-08**	**CALD1, CACNB2, TPM2, TPM1, DSTN, VCL, SORBS1, ANG, DMD, LMOD1, CNN1, RCSD1, KCNMA1, DIXDC1, ACTN1, SYNPO2, FLNC, PALLD, NEXN, FLNA, LSP1, SYNE1, TNS1, TAGLN, SVIL, MYH11, DST, MYLK, TMOD1**
**GO:0005509**	**Calcium ion binding**	**26**	**9.1**	**2.20E-04**	**2.20E-02**	**PAMR1, SNCA, C1S, MYL9, ANXA6, FAT3, CD93, PKD2, PLCD4, SVEP1, SPARCL1, SYT11, ACTN1, MGP, NRXN1, PCDH7, ITPR1, SLIT3, PLSCR4, FBLN5, CDH19, PLA2G2A, DST, CASQ2, SGCA, CDH11**
