# Supplementary material for: Identification of Key Biomarkers in Bladder Cancer: Evidence from a Bioinformatics Analysis
Source: Diagnostics (Basel). 2020 Jan 24;10(2):66. doi: 10.3390/diagnostics10020066 (PMC7168923; doi:10.3390/diagnostics10020066)
Supplement: Supplementary file 1 [file diagnostics-10-00066-s001.zip › Table S4.pdf]

Table S4. KEGG analysis. Analysis of total DEGs and the subgroups of up- and down-regulated DEGs. Numbers in brackets indicate count of DEGs.

ID	Description	Count in Gene set	Percent-age%	P-value	Benjamini	List of Genes	
						Up-regulated	Down-regulated
**Analysis of Total DEGs**							
hsa05200	Pathways in cancer	21	5	5.80E-03	1.70E-01	E2F2 CKS1B MMP9 BIRC5 TPM3 BAX CKS2 LAMC2 (8)	IL6 PTGER3 PTGS2 ROCK2 FGF9 RUNX1T1 FGF10 ZBTB16 FZD7 LAMA2 FOS PTCH1 FGF2 (13)
hsa04110	Cell cycle	17	4.1	2.40E-07	5.50E-05	CDK1 E2F2 TTK CHEK1 CDC20 PTTG1 MCM2 CDC25C MCM5 CCNB1 MAD2L1 CCNB2 PCNA BUB1 CCNA2 TFDP1 (16)	GADD45B (1)
hsa04510	Focal adhesion	13	3.1	1.10E-02	2.50E-01	PAK6 LAMC2 SPP1 (1)	ROCK2 ACTN1 FLNC FLNA VCL MYL9 LAMA2 ITGA8 ILK MYLK (10)
hsa04022	cGMP-PKG signaling pathway	12	2.9	4.20E-03	1.70E-01		KCNMA1 ATP2B4 ROCK2 PLN MRVI1 PDE5A CREB5 ATP1A2 MYLK ITPR1 KCNMB1 MYL9 (51)
hsa04024	cAMP signaling pathway	12	2.9	2.10E-02	3.10E-01	GRIN2D (1)	FOS PTGER3 ATP2B4 ROCK2 CAMK2G PLN ABCC4 CREB5 PTCH1 ATP1A2 MYL9 (11)
hsa05205	Proteoglycans in cancer	12	2.9	2.20E-02	3.10E-01	MMP9 PLAU (2)	GPC3 ROCK2 CAMK2G HBEGF PTCH1 FLNC FGF2 FZD7 FLNA ITPR1 (10)
hsa04810	Regulation of actin cytoskeleton	12	2.9	3.10E-02	3.60E-01	PAK6 DIAPH3 (2)	CHRM3 ROCK2 FGF9 ITGA8 FGF10 ACTN1 FGF2 MYLK MYL9 VCL (10)
hsa04114	Oocyte meiosis	11	2.6	9.30E-04	5.10E-01	CCNB1 CDK1 MAD2L1 CCNB2 BUB1 AURKA CDC20 PTTG1 CDC25C (9)	CAMK2G ITPR1 (2)
hsa04020	Calcium signaling pathway	11	2.6	2.60E-02	5.10E-02	GRIN2D (18)	PTGER3 ATP2B4 CHRM3 PDE1C CAMK2G PLN PLCD4 PTGFR MYLK ITPR1 (32)
hsa05410	Hypertrophic cardiomyopathy (HCM)	10	2.4	2.70E-04	3.30E-01	TPM3 (1)	IL6 ACTC1 DES DMD ITGA8 CACNB2 TPM2 TPM1 SGCA (9)
hsa05414	Dilated cardiomyopathy	10	2.4	4.80E-04	4.00E-01	TPM3 (1)	ACTC1 DES DMD PLN ITGA8 CACNB2 TPM2 TPM1 SGCA (9)
hsa04270	Vascular smooth muscle contraction	10	2.4	4.90E-03	3.00E-02		KCNMA1 ACTG2 ROCK2 CALD1 MRVI1 PLA2G2A MYLK ITPR1 KCNMB1 MYL9 (10)
hsa04261	Adrenergic signaling in cardiomyocytes	10	2.4	1.40E-02	3.50E-02	TPM3 (1)	ACTC1 ATP2B4 CAMK2G PLN CACNB2 CREB5 ATP1A2 TPM2 TPM1 (9)
**Analysis of Up-regulated DEGs**							
hsa04110	Cell cycle	16	12.1	9.1E-13	1.00E-10	CDK1, E2F2, TTK, CHEK1, CDC20, PTTG1, MCM2, CDC25C, MCM5, CCNB1, CCNB2, MAD2L1, PCNA, BUB1, CCNA2, TFDP1 *(16)*	
hsa04114	Oocyte meiosis	9	6.8	0.000015	8.40E-04	CCNB1, CDK1, MAD2L1, CCNB2, BUB1, AURKA, CDC20, PTTG1, CDC25C *(9)*	
hsa05203	Viral carcinogenesis	8	6.1	0.0046	8.90E-03	CDK1, HIST1H2BD, HIST1H2BK, BAX, CHEK1, CDC20, RANBP1, CCNA2 *(8)*	
hsa05166	HTLV-I infection	8	6.1	0.014	1.60E-02	E2F2, MAD2L1, BAX, PCNA, CHEK1, CDC20, RANBP1, PTTG1 *(8)*	
hsa04914	Progesterone-mediated oocyte maturation	7	5.3	0.00024	1.00E-01	CCNB1, CDK1, MAD2L1, CCNB2, BUB1, CDC25C, CCNA2 *(7)*	
hsa04115	p53 signaling pathway	6	4.5	0.00056	1.00E-01	CCNB1, CDK1, CCNB2, RRM2, BAX, CHEK1 *(6)*	
hsa05161	Hepatitis B	6	4.5	0.016	2.10E-01	E2F2, BAX, MMP9, PCNA, BIRC5, CCNA2 *(6)*	

Analysis of Down-regulated DEGs						
hsa05200	Pathways in cancer	13	4.5	3.90E-02	2.80E-01	IL6, PTGER3, PTGS2, ROCK2, FGF9, RUNX1T1, FGF10, ZBTB16, FZD7, LAMA2, FOS, PTCH1, FGF2 *(13)*
hsa04022	cGMP-PKG signaling pathway	12	4.2	8.20E-05	8.10E-03	KCNMA1, ATP2B4, ROCK2, PLN, MRVI1, PDE5A, CREB5, ATP1A2, MYLK, ITPR1, KCNMB1, MYL9 *(12)*
hsa04024	cAMP signaling pathway	11	3.8	2.20E-03	8.20E-02	FOS, PTGER3, ATP2B4, ROCK2, CAMK2G, PLN, ABCC4, CREB5, PTCH1, ATP1A2, MYL9 *(11)*
hsa04270	Vascular smooth muscle contraction	10	3.5	1.80E-04	8.70E-03	KCNMA1, ACTG2, ROCK2, CALD1, MRVI1, PLA2G2A, MYLK, ITPR1, KCNMB1, MYL9 *(10)*
hsa04020	Calcium signaling pathway	10	3.5	3.70E-03	9.90E-02	PTGER3, ATP2B4, CHRM3, PDE1C, CAMK2G, PLN, PLCD4, PTGFR, MYLK, ITPR1 *(10)*
hsa05205	Proteoglycans in cancer	10	3.5	7.60E-03	1.50E-01	GPC3, ROCK2, CAMK2G, HBEGF, PTCH1, FLNC, FGF2, FZD7, FLNA, ITPR1 *(10)*
hsa04510	Focal adhesion	10	3.5	9.20E-03	1.70E-01	LAMA2, ROCK2, ITGA8, ILK, ACTN1, FLNC, FLNA, MYLK, MYL9, VCL *(10)*
hsa04810	Regulation of actin cytoskeleton	10	3.5	1.00E-02	1.60E-01	CHRM3, ROCK2, FGF9, ITGA8, ACTN1, FGF10, FGF2, MYLK, MYL9, VCL *(10)*
hsa04010	MAPK signaling pathway	10	3.5	3.10E-02	2.60E-01	FOS, NTF3, FGF9, MAPT, CACNB2, FGF10, GADD45B, FLNC, FGF2, FLNA *(10)*
hsa05410	Hypertrophic cardiomyopathy (HCM)	9	3.1	5.40E-05	1.10E-02	IL6, ACTC1, DES, DMD, ITGA8, CACNB2, TPM2, TPM1, SGCA *(9)*
