# Supplementary material for: Identification of Key Biomarkers in Bladder Cancer: Evidence from a Bioinformatics Analysis
Source: Diagnostics (Basel). 2020 Jan 24;10(2):66. doi: 10.3390/diagnostics10020066 (PMC7168923; doi:10.3390/diagnostics10020066)
Supplement: Supplementary file 1 [file diagnostics-10-00066-s001.zip › Table S5.pdf]

Table S5. GO and KEGG pathway enrichment analysis of 14 hub genes in BC samples.
Numbers in brackets indicate count of hub genes.

ID	Description	Count in Gene set	Percentage%	P-value	List of Genes
**GOTERM_BP**					
GO:1902589	Single-organism organelle organization	12	85.7	4.40E-10	CCNB1, CDK1, KIF2C, KIF11, MAD2L1, CRYAB, AURKA, CDC20, UBE2C, TPM1, CASQ2, KIF20A *(12)*
GO:0051301	Cell division	10	71.4	4.20E-11	CCNB1, CDK1, KIF2C, KIF11, MAD2L1, AURKA, CDC20, UBE2C, CCNA2, KIF20A *(10)*
GO:1903047	Mitotic cell cycle process	10	71.4	2.00E-09	CCNB1, CDK1, KIF2C, KIF11, MAD2L1, AURKA, CDC20, UBE2C, CCNA2, KIF20A *(10)*
GO:0000278	Mitotic cell cycle	10	71.4	4.20E-09	CCNB1, CDK1, KIF2C, KIF11, MAD2L1, AURKA, CDC20, UBE2C, CCNA2, KIF20A *(10)*
GO:0022402	Cell cycle process	10	71.4	8.20E-08	CCNB1, CDK1, KIF2C, KIF11, MAD2L1, AURKA, CDC20, UBE2C, CCNA2, KIF20A *(10)*
GO:0007049	Cell cycle	10	71.4	5.00E-07	CCNB1, CDK1, KIF2C, KIF11, MAD2L1, AURKA, CDC20, UBE2C, CCNA2, KIF20A *(10)*
GO:0065009	Regulation of molecular function	10	71.4	4.80E-05	CCNB1, CDK1, MAD2L1, CRYAB, AURKA, CDC20, UBE2C, CCNA2, TPM1, CASQ2 *(10)*
GO:0007067	Mitotic nuclear division	9	64.3	2.20E-10	CCNB1, CDK1, KIF2C, KIF11, MAD2L1, AURKA, CDC20, UBE2C, CCNA *(9)*
GO:0000280	Nuclear division	9	64.3	2.40E-09	CCNB1, CDK1, KIF2C, KIF11, MAD2L1, AURKA, CDC20, UBE2C, CCNA2 *(9)*
GO:0048285	Organelle fission	9	64.3	4.00E-09	CCNB1, CDK1, KIF2C, KIF11, MAD2L1, AURKA, CDC20, UBE2C, CCNA2 *(9)*
**GOTERM_CC**					
GO:0005829	Cytosol	11	78.6	6.80E-05	CCNB1, CDK1, KIF2C, KIF11, MAD2L1, CRYAB, AURKA, CDC20, UBE2C, KPNA2, TPM1 *(11)*
GO:0015630	Microtubule cytoskeleton	9	64.3	9.60E-07	CCNB1, CDK1, KIF2C, KIF11, MAD2L1, CRYAB, AURKA, CDC20, KIF20A *(9)*
GO:000565	Nucleoplasm	9	64.3	1.50E-03	CCNB1, CDK1, CRYAB, AURKA, CDC20, UBE2C, KPNA2, CCNA2, KIF20A *(9)*
GO:0005819	Spindle	7	50	1.20E-07	CCNB1, CDK1, KIF11, MAD2L1, AURKA, CDC20, KIF20A *(7)*
GO:0099513	Polymeric cytoskeletal fiber	6	42.9	2.40E-04	CDK1, KIF2C, KIF11, AURKA, TPM1, KIF20A *(6)*
GO:0099512	Supramolecular fiber	6	42.9	2.40E-04	CDK1, KIF2C, KIF11, AURKA, TPM1, KIF20A *(6)*
GO:0000922	Spindle pole	5	35.7	4.50E-06	CCNB1, KIF11, MAD2L1, AURKA, CDC20 *(5)*
GO:0098687	Chromosomal region	5	35.7	1.90E-04	CCNB1, CDK1, KIF2C, MAD2L1, AURKA *(5)*
GO:0005874	Microtubule	5	35.7	3.50E-04	CDK1, KIF2C, KIF11, AURKA, KIF20A *(5)*
GO:0044427	Chromosomal part	5	35.7	4.90E-03	CCNB1, CDK1, KIF2C, MAD2L1, AURKA *(5)*
**GOTERM_MF**					
GO:0019899	Enzyme binding	8	57.1	2.50E-04	CCNB1, KIF11, AURKA, CDC20, UBE2C, KPNA2, CCNA2, KIF20A *(8)*
GO:0005524	ATP binding	6	42.9	5.50E-03	CDK1, KIF2C, KIF11, AURKA, UBE2C, KIF20A *(6)*
GO:0032559	Adenyl ribonucleotide binding	6	42.9	6.30E-03	CDK1, KIF2C, KIF11, AURKA, UBE2C, KIF20A *(6)*
GO:0030554	Adenyl nucleotide binding	6	42.9	1.30E-02	CDK1, KIF2C, KIF11, AURKA, UBE2C, KIF20A *(6)*
GO:0035639	Purine ribonucleoside triphosphate binding	6	42.9	1.40E-02	CDK1, KIF2C, KIF11, AURKA, UBE2C, KIF20A *(6)*
GO:0032550	Purine ribonucleoside binding	6	42.9	1.40E-02	CDK1, KIF2C, KIF11, AURKA, UBE2C, KIF20A *(6)*
GO:0001883	Purine nucleoside binding	6	42.9	1.40E-02	CDK1, KIF2C, KIF11, AURKA, UBE2C, KIF20A *(6)*
GO:0032549	Ribonucleoside binding	6	42.9	1.40E-02	CDK1, KIF2C, KIF11, AURKA, UBE2C, KIF20A *(6)*
GO:0001882	Nucleoside binding	6	42.9	1.50E-02	CDK1, KIF2C, KIF11, AURKA, UBE2C, KIF20A *(6)*
GO:0032555	Purine ribonucleotide binding	6	42.9	1.50E-02	CDK1, KIF2C, KIF11, AURKA, UBE2C, KIF20A *(6)*
**KEGG_PATHWAY**					
hsa04114	Oocyte meiosis	5	35.7	7.60E-06	CCNB1, CDK1, MAD2L1, AURKA, CDC20 *(5)*
hsa04110	Cell cycle	5	35.7	1.2E-05	CCNB1, CDK1, MAD2L1, CDC20, CCNA2 *(5)*
hsa04914	Progesterone-mediated oocyte maturation	4	28.6	1.6E-04	CCNB1, CDK1, MAD2L1, CCNA2 *(4)*
hsa05203	Viral carcinogenesis	3	21.4	2.8E-02	CDK1, CDC20, CCNA2 *(3)*
hsa04115	P53 signaling pathway	2	14.3	8.4E-02	CCNB1, CDK1 *(2)*
