# Supplementary figures and images for: Identification of Key Biomarkers in Bladder Cancer: Evidence from a Bioinformatics Analysis
Source: Diagnostics (Basel). 2020 Jan 24;10(2):66. doi: 10.3390/diagnostics10020066 (PMC7168923; doi:10.3390/diagnostics10020066)

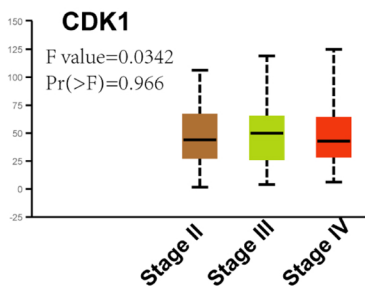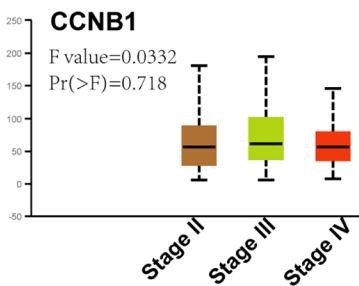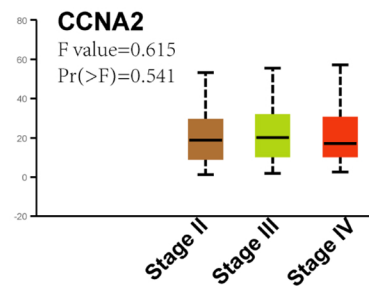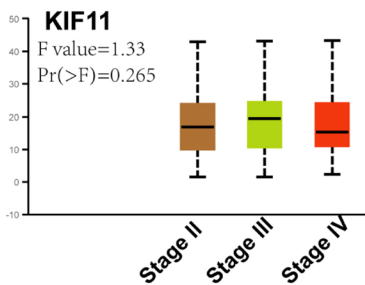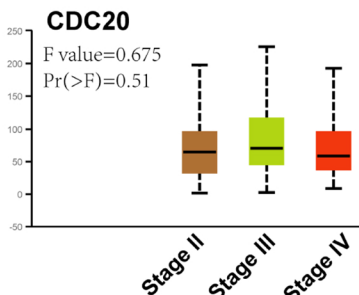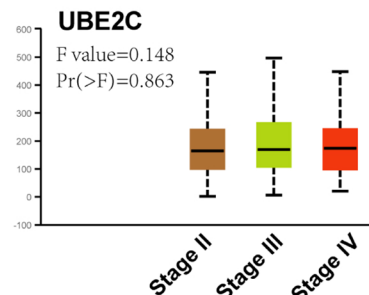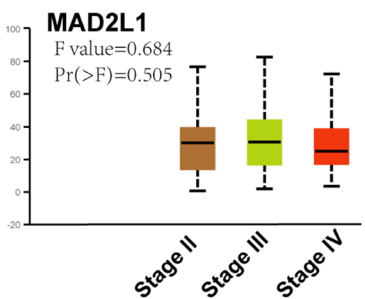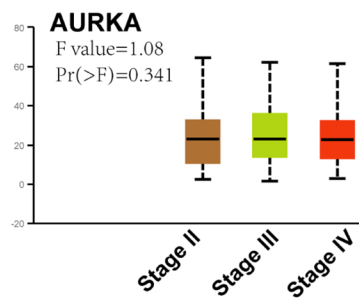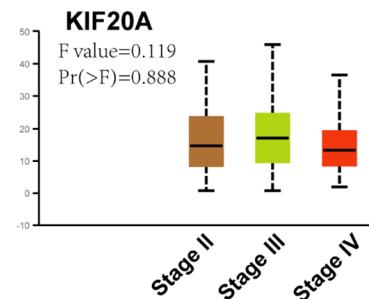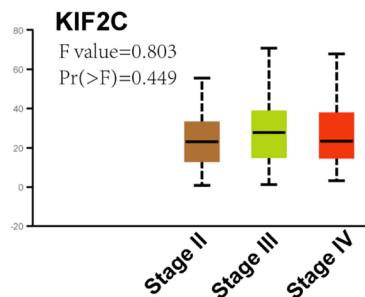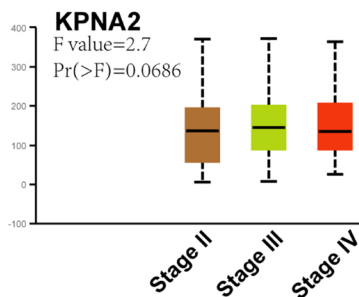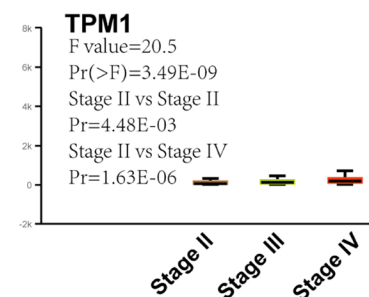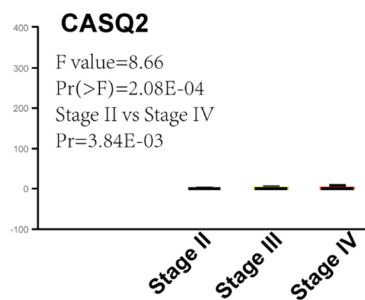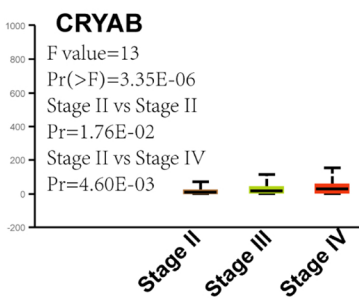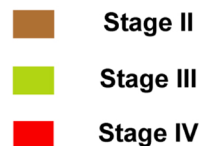

Supplement: Supplementary file 1 [file diagnostics-10-00066-s001.zip › Figure S2.pdf]
